# Efficient Coarse‐Grained Superplasticity of a Gigapascal Lightweight Refractory Medium Entropy Alloy

**DOI:** 10.1002/advs.202207535

**Published:** 2023-02-19

**Authors:** Yuefei Jia, Shiwei Wu, Yongkun Mu, Long Xu, Chang Ren, Kang Sun, Jun Yi, Yandong Jia, Wentao Yan, Gang Wang

**Affiliations:** ^1^ Institute of Materials Shanghai University Shanghai 200444 China; ^2^ Zhejiang Institute of Advanced Materials Shanghai University Jiashan 314100 China; ^3^ Department of Mechanical Engineering National University of Singapore Singapore 117575 Singapore

**Keywords:** coarse grain, high strain rate, high strength, lightweight medium entropy alloy, superplasticity

## Abstract

Superplastic metals that exhibit exceptional ductility (>300%) are appealing for use in high‐quality engineering components with complex shapes. However, the wide application of most superplastic alloys has been constrained due to their poor strength, the relatively long superplastic deformation period, and the complex and high‐cost grain refinement processes. Here these issues are addressed by the coarse‐grained superplasticity of high‐strength lightweight medium entropy alloy (Ti_43.3_V_28_Zr_14_Nb_14_Mo_0.7_, at.%) with a microstructure of ultrafine particles embedded in the body‐centered‐cubic matrix. The results demonstrate that the alloy reached a high coarse‐grained superplasticity greater than ≈440% at a high strain rate of 10^−2^ s^−1^ at 1173 K and with a gigapascal residual strength. A consecutively triggered deformation mechanism that sequences of dislocation slip, dynamic recrystallization, and grain boundary sliding in such alloy differs from conventional grain‐boundary sliding in fine‐grained materials. The present results open a pathway for highly efficient superplastic forming, broaden superplastic materials to the high‐strength field, and guide the development of new alloys.

## Graphical abstract

A coarse‐grained superplastic behavior is discovered in a lightweight refractory medium entropy alloy. A consecutively triggered "domino" superplastic mechanism is found to be a sequence of dislocation sliding, dynamic recrystallization, and grain boundary sliding, which can be consecutively triggered, like dominoes. This mechanism provides a method to develop the alloys with superplasticity without grain refinement. Therefore, superplastic alloys can be economically synthesized.

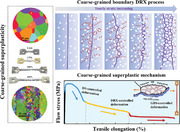

## 1 Introduction

Alloys exhibit superplastic states, allowing them to be deformed well beyond their usual breaking point under tensile strains greater than 300% without necking or fracturing at elevated temperatures above one‐half of their melting points.^[^ ^1^, ^2^, ^3^ ^]^ These studies have indicated that the superplasticity phenomenon is critical and essential for facilitating the manufacturing of complex, high‐quality components in many applications, such as aeronautics, space, transportation, and biomedical sectors.^[^ ^4^ ^]^ Until recently, most studies have focused on the superplasticity of conventional alloys, such as Mg‐based,^[^ ^2^, ^5^ ^]^ Al‐based,^[^ ^6^ ^]^ Zn‐based,^[^ ^7^ ^]^ Ni‐based,^[^ ^8^ ^]^ and Ti‐based alloys.^[^ ^9^, ^10^ ^]^ However, the wide application of most conventional superplastic alloys has been constrained by their poor strength, such as Mg‐based, Al‐based, and Zn‐based alloys,^[^ ^11^, ^12^, ^13^ ^]^ and the relatively long period required to reach the superplastic state, due to the low strain rates that can be applied on the order of 10^−3^ s^−1^.^[^ ^14^, ^15^, ^16^ ^]^ These issues have been recently addressed by developing fine/nanostructured face‐centered cubic (FCC) phase high‐/medium‐entropy alloys (HEAs/ MEAs), with excellent superplasticity, formed via a simple grain‐boundary siding (GBS) mechanism.^[^ ^17^, ^18^, ^19^, ^20^, ^21^, ^22^, ^23^, ^24^ ^]^ However, their strengths are still insufficient for many applications. Obtaining fine/nanocrystalline grains requires additional grain refinement processes, generally complex and high cost, such as high‐pressure torsion, friction stir processing or equal channel angular pressing, etc.,^[^ ^3^, ^18^, ^25^ ^]^ which significantly restricts the economic viability of these materials. Furthermore, low‐temperature superplastic forming is required to avoid grain growth during deformation, as a trade‐off, limiting the highest superplastic strain rates is necessary.^[^ ^26^ ^]^

These issues can be potentially addressed by developing high‐strength coarse‐grained superplastic alloys with grain sizes greater than 15 µm.^[^ ^16^, ^26^, ^27^, ^28^, ^29^ ^]^ In fact, high ductility at strain rates on the order of 10^−2^ s^−1^ has been demonstrated for a commercial coarse‐grained Al–Mg alloy with low strength.^[^ ^16^ ^]^ Nonetheless, this task has been quite challenging because coarse‐grained alloys rarely exhibit superplasticity at high strain rates due to their poor GBS ability.^[^ ^3^, ^14^, ^30^ ^]^ Coarse‐grained superplasticity is generally considered to be a mechanism of solute drag creep, which produces a tensile elongation of ≈200%, regardless of grain size.^[^ ^31^ ^]^ The elongations can be further enhanced by dynamic recovery or dynamic recrystallization (DRX).^[^ ^16^, ^26^, ^27^, ^29^, ^32^ ^]^ However, high‐/medium‐ entropy solid solutions are generally considered to be free of specific solutes and solvents, distinguished from conventional single principal solid solutions.^[^ ^33^, ^34^ ^]^ As a result, coarse‐grained HEA/MEAs are not suitable for conventional coarse‐grained superplasticity mechanisms where the solute drag creep is dominant.

Another disadvantage of the reported superplastic HEA/MEA materials is that they tend to have relatively high densities, which limits their application, such as structural aeronautical materials. An emerging class of structural materials denoted as Light Weight HEAs/MEAs (LWHEAs/MEAs) has been developed with both high strength and low density in the range of 1.5–6.5 g cm^−3^;^[^ ^35^, ^36^, ^37^, ^38^, ^39^ ^]^ therefore, they have considerable potential for use as next‐generation aeronautical structural materials. However, most LWHEA materials are poorly suited for machining into complex shapes due to their limited ductility at ambient temperatures, and their superplasticity at elevated temperatures has been poorly documented.

In this work, we address the above‐discussed issues by investigating the superplasticity of a lightweight refractory MEA (LRMEA) with the formula Ti_43.3_V_28_Zr_14_Nb_14_Mo_0.7_, with the mixing entropy of ≈1.32*R* (*R* = 8.314 J mol^−1^ K^−1^ is the gas constant), exhibiting high strength and coarse grains here, which was denoted as HC‐LRMEA. The primary strengthening mechanism in these LRMEAs is derived from high solid solution strengthening due to a high lattice distortion and minor particle strengthening.^[^ ^40^ ^]^ The Hall–Petch slope of the LRMEAs is low, which is ≈7.9 MPa$\sqrt{mm}$. Therefore, the yield strength is not significantly affected by a grain size^[^ ^40^, ^41^ ^]^ when the LRMEAs are coarse grained. Furthermore, body‐centered cubic (BCC) RHEA/MEAs exhibit a steady‐state flow at nearly constant flow stress at high temperatures.^[^ ^42^, ^43^ ^]^ The steady‐state flow capability is crucial for superplastic deformation. Therefore, we believe that the BCC‐based LRMEA has a potentially coarse‐grained superplasticity and does not significantly sacrifice strength due to grain coarsening. So far, to the best of our knowledge, the coarse‐grained superplasticity at high strain rates (≥10^−2^ s^−1^) has not been reported for other gigapascal HEAs/MEAs, especially for LRMEA with the BCC‐based microstructure. The results of this study demonstrate that the HC‐LRMEA could attain superplastic tensile strain greater than ≈440% at a temperature of 1173 K (i.e., 0.55 times the melting temperature *T*_m_ of 2158 K) and the superplastic state could be efficiently attained at a high strain rate of 10^−2^ s^−1^. In contrast to the conventional GBS mechanism leading to superplasticity in fine‐grained materials, the superplasticity of this alloy was demonstrated to occur via the consecutive sequence of three primary mechanisms. These mechanisms operate successively with increasing specimen elongation, including dislocation slip (DS) inside the coarse grains at low specimen elongation, and dynamic recrystallization (DRX) at the coarse‐grain boundary areas under moderate specimen elongation, producing fine‐grained particles that facilitate GBS at superplastic elongations. This superplasticity process, denoted herein as the “domino” superplastic mechanism, consists of three consecutively triggered primary mechanisms. As a result, it would allow superplastic materials to be more economically viable by enabling the rapid formation of superplasticity without introducing grain refinement processes.

## 2 Results and Discussion

### 2.1 Initial Microstructures and Mechanical Performance

**Figure 1:**
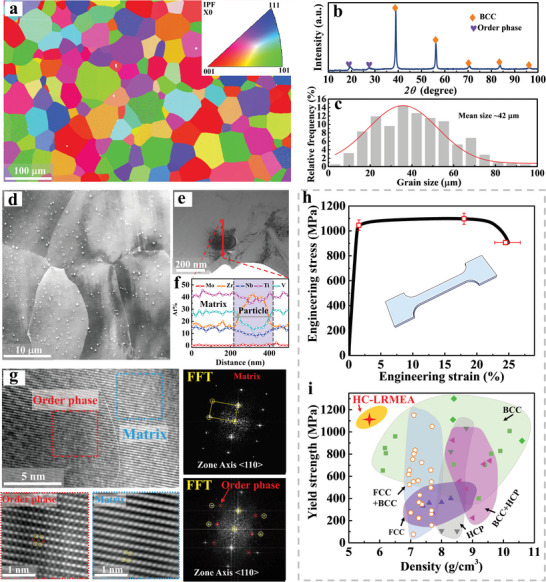
Microstructures and mechanical performance of the HC‐LRMEA samples. a) EBSD IPF map; b) XRD pattern; c) grain size distribution calculated from the three different IPF maps; d) BSE image showing distinct particles inside the grains; e) STEM image exhibiting the morphology of the order‐phase particles; f) STEM‐EDS line profiles presenting the elemental distribution across the BCC matrix and order‐phase particle; g) HRTEM and corresponding FFT images of the BCC matrix and order phase along the <110> direction; h) tensile engineering stress‐engineering strain curve at room temperature; i) maps of yield strength versus density of the ductile BCC, FCC, HCP, BCC+HCP, FCC+BCC HEAs reported previously at room temperature.

The initial microstructures of the HC‐LRMEA samples prior to tensile testing were analyzed, as shown in **Figure** **1**a–g. A representative EBSD inverse pole figure (IPF) is presented in Figure 1a, including an IPF map indicative of the crystal orientation shown in the inset. Note that the initial HC‐LRMEA sample exhibited a typical polycrystalline microstructure composed of recrystallized and equiaxed grains. The analysis presented in Figure 1c, indicated that the mean grain size was ≈42 µm. The XRD pattern illustrated in Figure 1b for the initial HC‐LRMEA sample is indicative of a dual‐phase composition with BCC matrix and order‐phase particles. The dual‐phase morphology is better characterized by the backscattered electron image presented in Figure 1d and the scanning transmission electron microscopic (STEM) image illustrated in Figure 1e. These results demonstrate that the submicron‐size order‐phase particles are uniformly distributed in the BCC‐phase matrix, with sizes on the order of 50–500 nm with a volume fraction of ≈7%. In addition, the energy‐dispersive X‐ray spectroscopy (EDS) results obtained for a single grain in conjunction with the STEM image in Figure 1f indicate that elements Ti, V, Zr, Nb, and Mo are homogeneously distributed in the BCC‐phase matrix, while the element Zr is enriched in the order‐phase region of the grain (subsequently known as Zr‐rich particles). The presence of a mixed BCC‐phase matrix and secondary ordered‐phase structure is further confirmed by the high‐resolution transmission electron microscopy (HRTEM) and corresponding fast Fourier transform (FFT) images presented in Figure 1g.

Figure 1h presents the uniaxial tensile engineering stress‐strain curve of the annealed HC‐LRMEA at room temperature with a strain rate of 10^−3^ s^−1^, which exhibits an ultrahigh tensile strength and high ductility at room temperature. Specifically, a yield strength (*σ*_0.2_) of ≈1045 ± 43 MPa, an ultimate strength, UTS, of ≈1097 ± 45 MPa, and elongation of ≈24.5 ± 2.3% (uniform elongation of ≈18 ± 1.5%) are observed. To demonstrate the performance advantage of the HC‐LRMEA, a comparison of the tensile properties is conducted, as shown in Figure 1i. The MEAs or HEAs were classified into six types, including FCC, BCC, hexagonal‐close packed (HCP), and dual‐phase structures.^[^ ^33^, ^44^, ^45^, ^46^, ^47^, ^48^, ^49^, ^50^, ^51^, ^52^, ^53^ ^]^ Overall, the FCC HEAs/MEAs generally show low strength but high density, and the BCC HEAs/MEAs exhibit high yield strengths, especially the ordered oxygen complexes strengthen RHEAs,^[^ ^53^ ^]^ but a wide range of densities. The FCC + BCC HEAs/MEAs exhibit higher strength and lighter density than the pure FCC MEAs, generally due to the high content of aluminum elements. The HCP HEAs/MEAs and BCC + HCP dual‐phase alloys occupy the middle region. In Figure 1i, obviously, HC‐LRMEA occupies an unexplored space, as it is both light (<6 g cm^−3^) and strong (>1 GPa) and shows sufficient ductility (>20%) at room temperature.

### 2.2 Superplastic Behaviors

**Figure 2:**
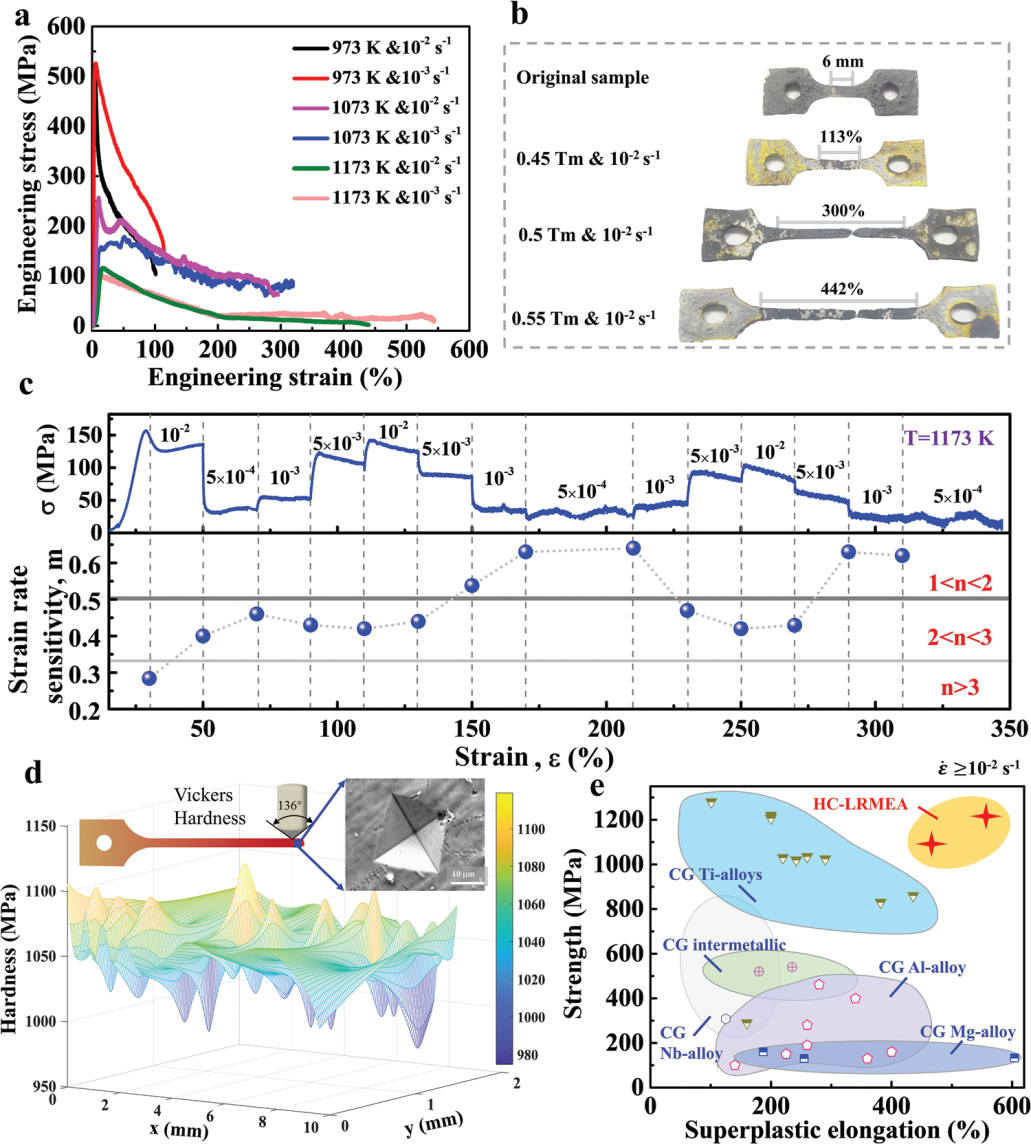
Superplastic behaviors of the HC‐LRMEA samples. a) engineering stress‐strain curves obtained under tensile loading at temperatures of 973, 1073, and 1173 K and strain rates of 10^−3^ and 10^−2^ s^−1^; b) images of representative specimens fractured under temperatures of 973, 1073, and 1173 K and a strain rate of 10^−2^ s^−1^; c) stress–strain, *σ–ε*, curves and strain‐rate sensitivity (*m*) values obtained from strain‐rate jump tests conducted at 1173 K with 4 different strain rates of 5 × 10^−4^, 1 × 10^−3^, 5 × 10^−3^, and 1 × 10^−2^ s^−1^, where *n* = 1/*m* is the stress exponent; d) the hardness distribution of the sample after coarse‐grained superplastic deformation; the inset in (d) illustrates the hardness test and morphology of hardness indentation; e) yield strength versus superplastic elongation at high strain rates (10^−2^ s^−1^) of coarse‐grained superplastic magnesium alloys,^[^ ^25^, ^26^, ^58^, ^59^, ^60^, ^61^, ^62^ ^]^ coarse‐grained superplastic aluminum alloys,^[^ ^28^, ^63^, ^64^, ^65^, ^66^, ^67^, ^68^, ^69^, ^70^, ^71^, ^72^ ^]^ coarse‐grained niobium alloy,^[^ ^73^ ^]^ coarse‐grained superplastic intermetallics,^[^ ^74^, ^75^, ^76^ ^]^ and coarse‐grained superplastic titanium alloys.^[^ ^9^, ^77^, ^78^, ^79^, ^80^, ^81^, ^82^, ^83^, ^84^ ^]^

The representative engineering stress‐strain curves obtained for the HC‐LRMEA specimens under tensile loading at temperatures of 973, 1073, and 1173 K and strain rates of 10^−3^ and 10^−2^ s^−1^ are presented in **Figure** **2**a. The specimens obtained after fracture under different temperatures at a high strain rate of 10^−2^ s^−1^ are displayed in Figure 2b. Based on the measured elongation at fracture, the fractured specimens presented in Figure 2b show representative elongation values of ≈113% at 973 K (0.45*T*_m_), ≈300% at 1073 K (0.5*T*_m_), and ≈442% at 1173 K (0.55*T*_m_). The results indicate that the total elongation of the specimen loaded at 0.45*T*_m_ exhibited no obvious superplasticity, while the degree of elongation increased substantially with increasing temperature to 0.5*T*_m_, and apparent superplasticity is observed at 0.55*T*_m_. For comparison, note that specimens fractured under a significantly reduced strain rate of 10^−3^ s^−1^ provided elongations at fracture of 100% at 973 K, 320% at 1073 K, and 549% at 1173 K, which are quite similar to those observed at a much higher strain rate of 10^−2^ s^−1^. These results demonstrate that HC‐LRMEA possessed superplasticity at a high strain rate of 10^−2^ s^−1^ under temperatures greater than 0.5*T*_m_.

Strain‐rate sensitivity, *m*, is a critical parameter used to characterize the ability of metals to resist necking during deformation at high temperatures and is defined as^[^ ^54^ ^]^

(1) m=∂lnσ/∂lnε

where *σ* is the flow stress and *ε* is the plastic strain rate.^[^ ^14^ ^]^ In addition, the stress exponent *n* = 1/*m* has been generally employed to characterize high‐temperature deformation mechanisms. The values of *m* (and, hence *n*) were calculated for the HC‐LRMEA samples based on the results of the strain‐rate jump tests conducted under a temperature of 1173 K at 4 different strain rates of 10^−2^, 5×10^−3^, 10^−3^, and 5×10^−4^ s^−1^. The obtained values of *σ* are plotted as a function of *ε* in Figure 2c, and the calculated values of *m* are plotted along with the corresponding ranges of *n*. Notably, *m* varies from 0.29 to 0.64 over the full range of *ε*. The observed deformation could be divided into three stages according to the ranges *n* > 3, 2 < *n* ≤ 3, and 1 < *n* ≤ 2, which could be respectively defined as Stage I (*m* < ≈0.33), where *ε* < ≈30%, Stage II (0.33 < *m* < 0.5), with *ε* ranging from ≈50% to ≈145%, and Stage III (*m* > ≈0.5), with *ε* ranging from 145% to 315%. Although when the *ε* value is in the range from 250 to 280%, the *m* value is 0.45–0.5, which would have belonged to Stage II, for facilitating the exploration of the overall deformation mechanism, we attribute this plastic deformation section to be the third stage as well.

The strength of the HC‐LRMEA after superplastic deformation at 1173 K with the strain rate of 10^−2^ s^−1^ is demonstrated by a hardness test, as shown in Figure 2d. The hardness test results reveal that HC‐LRMEA retains an extremely high hardness level after superplastic deformation, with an average hardness value of ≈310 HV. By converting this hardness to the strength, a formula of HV ≈3*σ*_y_^[^ ^55^ ^]^ is used to characterize the strength, which indicates that the strength of HC‐LRMEA is still higher than 1 GPa after superplastic deformation (Figure 2d). In addition, HC‐LRMEA is compared with other coarse‐grained superplastic alloys, including coarse‐grained superplastic magnesium alloys,^[^ ^26^, ^27^, ^56^, ^57^, ^58^, ^59^, ^60^ ^]^ coarse‐grained superplastic aluminum alloys,^[^ ^29^, ^61^, ^62^, ^63^, ^64^, ^65^, ^66^, ^67^, ^68^, ^69^, ^70^ ^]^ coarse‐grained niobium alloy,^[^ ^71^ ^]^ coarse‐grained superplastic intermetallics,^[^ ^72^, ^73^, ^74^ ^]^ and coarse‐grained superplastic titanium alloys,^[^ ^9^, ^75^, ^76^, ^77^, ^78^, ^79^, ^80^, ^81^, ^82^ ^]^ Superplastic elongation at high strain rates (≥10^−2^ s^−1^) versus strength is plotted in Figure 2e. The strength values of the coarse‐grained superplastic magnesium and aluminum alloys are relatively low, making them difficult to use in some high‐strength conditions. Moreover, the coarse‐grained superplastic intermetallic compounds show relatively higher strength, but hardly reach the gigapascal level (Figure 2e). Some coarse‐grained superplastic titanium alloys can achieve high strengths, even over 1 GPa, but rarely maintain high strain rates and coarse‐grained superplasticity simultaneously. HC‐LRMEA is located in the upper right corner of the superplastic elongation‐strength diagram and exhibits gigapascal strength as well as excellent coarse‐grained superplastic deformability (Figure 2e).

Due to the fast recovery of BCC alloys, it is typically rare for BCC alloys to exhibit coarser‐grained superplastic behavior, especially in high‐strength refractory alloys.^[^ ^83^, ^84^ ^]^ In this study, coarse‐grained superplasticity was achieved in the ultrastrong LRMEA with a BCC structure at a high strain rate. In addition, after superplastic deformation, the HC‐LRMEA samples still retain their strength at a high level (>1 GPa) (Figure 2d). Thus, there is a remarkable combination of gigapascal strength and high‐strain‐rate coarse‐grained superplastic deformability in the BCC‐based HC‐LRMEA.

### 2.3 Microstructure Evolution

**Figure 3:**
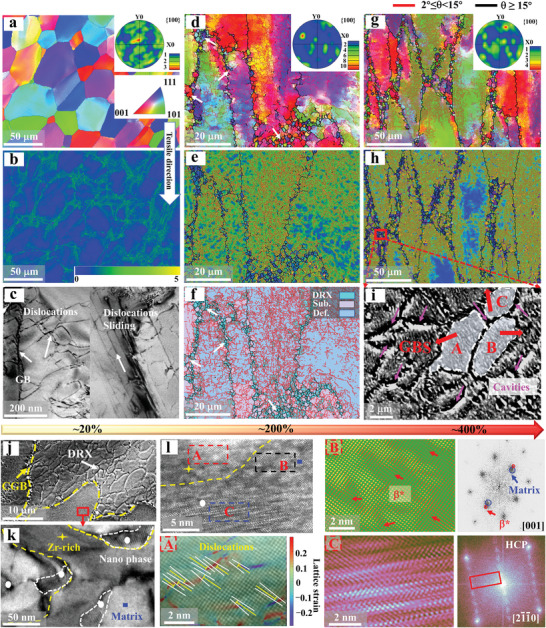
Microstructural evolution of the deformed specimens obtained at Stage I (*ε* ≈ 20%), Stage II (*ε* ≈ 200%), and Stage III (*ε* ≈ 400%) under a temperature of 1173 K and strain rate of 10^−2^ s^−1^. a,d,g) IPF maps, where the insets represent the crystal orientation; b,e,h) KAM maps corresponding to the IPF maps in (a), (d), and (g). c) TEM‐BF image at Stage I; f) DRX map of (d); i) SEM image corresponding to the selected area in (h) exhibiting regions associated with grain boundary sliding (GBS) and cavities marked by pink arrows; j) SEM image at coarse‐grain boundary after superplastic deformation; k) TEM‐BF image presenting many nanophases at the Zr‐rich particle boundary; l) HRTEM image indicating the Zr‐rich particle and ribbon nanophase; region “A” located in the Zr‐rich particle; region “B” located in the matrix; region “C” located in the ribbon nanophase.

The mechanisms responsible for the uncommon superplasticity observed for the HC‐LRMEA samples at a high strain rate are evaluated by examining the microstructures of the specimens obtained at three superplastic deformation strains under a temperature of 1173 K and strain rate of 10^−2^ s^−1^. Representative IPF maps are obtained during *ε* ≈ 20%, *ε* ≈ 200%, and *ε* ≈ 400%, as presented in **Figure** **3**a,d,g, respectively, where the inset shows the crystal orientation. In addition, the kernel average misorientation (KAM) maps corresponding to the IPF maps shown in Figure 3a,d,g are also shown in Figure 3b,e,h, respectively.

At a deformation strain of ≈20% (during Stage 1), elongated coarse grains are observed along the tensile direction, and no DRX grains are observed at the grain boundary areas (Figure 3a), while the corresponding KAM map indicates that substantial local strains are concentrated near the grain boundary areas (Figure 3b). Moreover, the TEM bright‐field image presented in Figure 3c exhibited an accumulation of dislocations at the grain boundaries and DS inside the grains during Stage I (Figure S7, Supporting Information). The mean geometrically necessary dislocations (GND) are also evaluated at about 1.8×10^14^ m^−2^ at the coarse‐grain boundary areas (Figure S5d, Supporting Information), which is much higher than the intragranular GND of ≈8.9×10^12^ m^−2^ (Figure S5a, Supporting Information). Dislocations at the coarse‐grain boundary areas accumulated at such a high density that they ended up in a state of high energy instability. Furthermore, numerous submicron particles at the grain boundary areas also facilitate the formation of subgrains or recrystallized grains, with the conjunction of dislocations and sub‐micrometer particles (Figure S8, Supporting Information).

With a superplastic strain of ≈200% (during Stage II), the coarse grains are further deformed in the tensile directions (Figure 3d,e), and many fine DRX grains are visible at the boundaries of the coarse grains, as marked by the white arrows in Figure 3d, and more clearly illustrated by the DRX map in Figure 3f. The new fine DRX grains are measured to be 1–7 µm in size (Figure S6b, Supporting Information). And the corresponding volume fraction for the fine DRX grains is about ≈12.5% (Figure S6a, Supporting Information). Interestingly, the corresponding KAM map (Figure 3e) exhibits reduced the local strain at the boundary areas of the coarse grains in conjunction with the formation of the fine DRX grains. Accordingly, intergranular GND is estimated at ≈6×10^13^ m^−2^ (Figure S5e, Supporting Information), a significant decrease compared to 20% deformation, which indicates that the accumulation of dislocations at the coarse‐grain boundary areas promoted DRX, and the recrystallization process consumes the excess energy induced by this dislocation accumulation.^[^ ^6^, ^85^ ^]^ In addition, Figure 3e indicates that severe local strain, namely, the GND concentrations, formed at the low‐angle boundaries within the coarse grains. The phenomenon means that the deformation of the original coarse grains is still dominated by dislocation slip, along with an increase in the dislocation density of ≈2.4×10^15^ m^−2^ (Figure S5b, Supporting Information), which is associated with the presence of numerous submicron particles (Figures S2 and S8c, Supporting Information). This is the result of particle‐dislocation interactions, and the mass subgrains and minor DRX grains forming within the original coarse grains (Figure S8c,d, Supporting Information). It is also consistent with the precipitates promoting subcrystallization and recrystallization in Al–Mg alloys and titanium alloys.^[^ ^6^, ^86^, ^87^ ^]^

When the superplastic deformation reaches 400% (during Stage III), the coarse grains are elongated quite severely. The area fraction of fine DRX grains at the coarse‐grain boundary areas (Figure S6a, Supporting Information) increased to ≈15.6%. The DRX grains remain in the range of 1.5–8.5 µm during the process (Figure S6b, Supporting Information). The DRX grains has no significant growth due to the high strain rates because that provide an insufficient time for the growth. In addition, the SEM image in Figure 3i corresponding to the selected area marked in Figure 3h exhibits the region associated with GBS along the boundaries of the coarse grains, where the fine DRX grains were generated. And pink arrows mark the cavities in Figure 3i. Of note, the adjacent fine DRX grains denoted as A, B, and C are separated by the cavities. These results demonstrate that superplastic deformation occurs within the coarse‐grain regions during Stage III via creep, which is controlled by the gliding of dislocations, while GBS occurs in the fine‐grain DRX regions that formed at the coarse‐grain boundary areas.

After the fracture, numerous fine DRX grains at the coarse grain boundary are produced (Figure 3j). And the Zr‐rich precipitates are connected to form an irregular shape (Figure 3k; Figure S2, Supporting Information). In addition, many nanophases could be observed at the boundary of the Zr‐rich particles. High‐resolution observations are obtained for a clearer view of the Zr‐rich particles and nanophases, and the corresponding HRTEM image along the [001] direction is shown in Figure 3l. There are three regions, where “A” is the inner region of the Zr‐rich particles, “B” is the boundary region near the tip of the nanophase, ad “C” is the region where the nanophase is located. Region “A” demonstrates that the Zr‐rich particles also underwent synergistic plastic deformation at high temperatures, causing severe lattice strain and the formation of numerous dislocations (Figure 3“A”). Region “B” displays several unknown phases (marked as β*) with sizes of 1–5 nm in this region (Figure 3“B”). The HRTEM image (Figure 3“C”) and corresponding FFT pattern indicate the ribbon nanophase with an HCP structure. The nanostructure phases that are formed from superplastic deformation allow deformation storage energy to be released from their confinement while at the same time promoting the DRX process.

Taking all these observations into account, we could surmise that the flow stress is relaxed throughout the deformation process via a sequence of DS within the coarse grains, DRX process at the coarse‐grain boundary areas and GBS processes at fine DRX grains region.

### 2.4 Superplasticity Mechanism

Consequently, we attribute the coarse‐grained superplasticity at the high strain rate to a consecutively triggered deformation mechanism, namely, “domino” superplastic mechanism in the current HC‐LRMEA (Figure S10, Supporting Information). Plastic deformation is dominated by DS during Stage I (*m* < 0.33 and *n* > 3). An increasing number of dislocations accumulate at the coarse‐grain boundary areas and ultrafine particles with increasing deformation, and this promotes DRX during Stage II (0.33 < *m* < 0.5 and 2 < *n* ≤ 3). Finally, superplastic deformation is dominated by GBS in the fine‐grain DRX regions during Stage III (*m* > 0.5 and 2 ≤ *n* < 1). As opposed to conventional coarse‐grained alloys deformation dominated by solute drag creep, this deformation is a multi‐deformation mechanism, which is why gigapascal LRMEA with BCC‐based coarse grains exhibit excellent superplastic deformation ability at high strain rates (≥10^−2^ s^−1^).

Specifically, high‐temperature deformation is associated with three distinct mechanisms that can occur at the atomic level, including (a) slippage by the movement of dislocations, (b) sliding of adjacent grains along grain boundaries (i.e., GBS), and (c) directional diffusional flow.^[^ ^14^ ^]^ The fully coarse‐grained structure of the HC‐LRMEA specimen during Stage I substantially inhibits the occurrence of GBS at the high applied strain rate on the order of 10^−2^ s^−1^. Moreover, the high strain rate provides insufficient time for directional diffusional flow. The stress exponent values are in a range of 3.3–4, suggesting that dislocation‐controlled creep^[^ ^88^ ^]^ was the dominant mechanism at Stage I. It was further supported by high‐density dislocations produced at this stage. Moreover, the ultrafine particles in the HC‐LRMEA produce high deformation stress (>100 MPa), which is sufficient to overcome the solute drag effect. The TEM observation demonstrates dislocations sliding dominate the dislocation‐controlled creep at Stage I (Figure 3c; Figure S7, Supporting Information). Increasing deformation induces the movement of an increasing number of dislocations that piles up at the boundaries of the coarse grains, as well as the particles, increasing the internal elastic strains at the grain boundaries and providing an abundance of nucleation sites^[^ ^89^ ^]^ for the formation of fine DRX grains.

Accordingly, DRX plays a significant role in the deformation observed during Stage II. In the current HC‐LRMEA, there are a variety of particles, including Zr‐rich and HCP particles (Figure 3k,l; Figure S2, Supporting Information), which made the dislocations more stable even at high temperatures (without the particles, massive dislocations would be unsustainable due to dynamic recovery). Meanwhile, the grain boundaries also play a crucial role in the recrystallization process as high‐energy defects. However, at the coarse‐grain boundary areas, DRX behavior induced by particles, the dislocations and grain boundaries in this HC‐LRMEA are distinguished from grain boundary migration dominated DRX in single‐phase alloys, and the presence of particles and dislocations would accelerate the DRX process, even at a high strain rate of 10^−2^ s^−1^.

**Figure 4:**
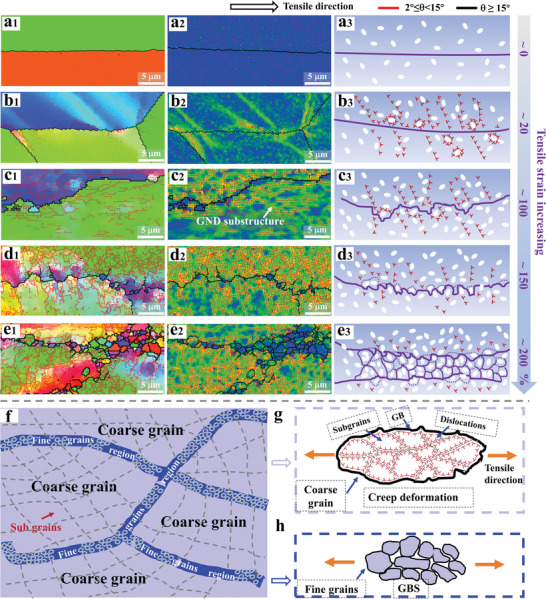
a–e) EBSD characterization and schematic illustrations of the deformation process observed for coarse‐grained HC‐LRMEA specimens with increasing tensile strains of 0, ≈20%, ≈100%, ≈150%, and ≈200%: 1 IPF maps; 2 KAM maps; 3 schematic illustrations indicative of the operative deformation mechanisms. f) schematic illustration showing the subgrains in the coarse‐grain region and the fine DRX grain band; g) schematic illustration showing the deformation mechanism in the coarse grains; h) schematic illustration showing the grain‐boundary sliding in the fine DRX grain band.

The overall deformation process observed for the HC‐LRMEA specimens is illustrated in detail in **Figure** **4**. The EBSD IPF and KAM maps present a straight grain boundary without any local strains or low‐angle components of HC‐LRMEA before deformation in Figure 4a1,a2. This condition is further highlighted by the schematic representation offered in Figure 4a3. The corresponding conditions observed with increasing tensile strain in the downward direction are illustrated in Figure 4b1–b3, where DS is initiated, and dislocations start to pile up at the grain boundary. Moreover, the precipitations near the grain boundary also accumulate high‐density dislocations. As a result, the original straight grain boundary becomes bent. The cause of this condition is the high temperature, which softens the grain boundary, and a significant stress difference forms between the inside and outside regions of the coarse grain boundary. The conditions under further increasing strain are illustrated in Figure 4c1–c3, where the grain boundary becomes deformed locally due to differences in local dislocation densities between the two sides of the grain boundary.

Additionally, the discontinuous pinning of the grain boundaries by the Zr‐rich particles also causes the grain boundaries to be deformed locally.^[^ ^89^, ^90^ ^]^ Furthermore, numerous high GND substructures formed around the grain boundaries due to the Zr particles locking massive dislocations during the deformation process. The conditions at even higher strains are illustrated in Figure 4d1–d3, where more GND substructures are visible. Due to the high diffusivity of atoms in the BCC crystals^[^ ^91^, ^92^ ^]^ and the dislocation pinning effect of the Zr‐rich particles, most of the GND substructures are transformed into low‐angle grain boundaries, namely, sub‐grain boundaries that are connected to the primary grain boundaries, reducing the free energy of the system. These connected subgrains then grew into fine DRX grains. A discontinuous dynamic recrystallization (DDRX) mechanism^[^ ^90^ ^]^ dominates the formation of the first layer of DRX grains, in which the grain boundary bulging out is a significant character. In addition, the high‐density dislocations and the precipitates distributed at the original coarse grain boundary can promote DDRX nucleation.

Subsequently, strain incompatibilities between the original grains and the softer, finer DRX grains generated additional subgrain boundaries and the growth of a second layer of the fine DRX grains. Finally, additional layers of DRX grains developed with further increasing strain, as illustrated in Figure 4e1–e3. Distinguishing from the first layer, the second and the expansion layers depend on a continuous dynamic recrystallization (CDRX) mechanism.^[^ ^90^ ^]^ The KAM maps in Figure 4d2,e2 demonstrate that the DRX process consumed high‐density dislocations at the primary grain boundary, reducing the local strain concentration in that location. During the superplastic deformation process, numerous dislocations are absorbed by DRX, which prevents necking and premature fracture caused by stress concentrations in the grain boundary areas. Moreover, recrystallization produces abundant fine DRX grains at the coarse grain boundaries, which provides a necessary condition for GBS.

The DRX process releases stress and strain concentrations to a large extent, thus, avoiding premature necking and fracture.^[^ ^93^, ^94^, ^95^ ^]^ The DRX nucleation begins preferentially at the trigonal grain boundary areas where the stress concentration is high, and belongs to a high‐energy region. At the low strain, many fine grains are produced by the DRX at the trigonal grain boundary areas, but only minor DRX grains are present at the straight grain boundary areas (Figures S10a,a1, Supporting Information). With deforming, the coarse grains are significantly elongated, and more and more DRX grains are formed at the straight grain boundary areas (Figure S10b,b1, Supporting Information). Eventually, numerous fine DRX grains are formed at the trigonal and straight grain boundary areas, which conforms a necklace structure of fine grains surrounding the coarse grains, as shown in Figure 4f. The necklace structures facilitate the GBS process in the fine‐grain DRX regions. Accordingly, GBS serves as the primary superplastic deformation mechanism for these regions, as verified for the BCC‐phase refractory HEAs.^[^ ^95^, ^96^ ^]^ Meanwhile, dislocation‐controlled creep is the primary superplastic deformation mechanism within the coarse grains (Figure 4g). Additionally, the refined and equiaxed DRX grains of the HC‐LRMEA could create favorable conditions for good superplasticity, as is found for conventional alloys.^[^ ^17^, ^93^ ^]^ Finally, because *m* > 0.5 during Stage III and the activation energy *Q* is 314.6 kJ mol^−1^ (Figure S1b, Supporting Information), this indicates that creep and diffusion jointly controlled the GBS process. Thus, GBS dominates the superplastic deformation observed in Stage III.

The relationship between the strain rate and superplastic deformation at 973, 1073, and 1173 K with different strain rates of 10^−2^ and 10^−3^ s^−1^ in the coarse‐grained LRMEAs is characterized in Figure 2 and Figure S4 in the Supporting Information. It can be seen that, when the temperatures are less than 1173 K, the plastic strains of HC‐LRMEA deformed at a high strain rate (10^−2^ s^−1^) and a low strain rate (10^−3^ s^−1^) are almost same (Figure 2a; Figure S4, Supporting Information). Actually, the low temperature can significantly prohibit the dynamic recrystallization that dominates the coarse‐grained superplasticity.^[^ ^97^, ^98^ ^]^ Thus, the strain‐rate sensitivity of the plasticity in the HC‐LRMEA is not significantly. Nevertheless, when the superplastic deformation occurred at 1173 K, the low strain rate (10^−3^ s^−1^) deformation can enhance the elongation of HC‐LRMEA to be larger than the high strain rate (10^−2^ s^−1^) deformation case by ≈20% (Figure 2a; Figure S4, Supporting Information). Numerous fine grains are produced at the grain boundaries due to the dynamic recrystallization (Figure 3f). In addition, as compared with the case at the high strain rates, the grain boundary slip at the low strain rates is more easier to be activated in the fine grain regions than that in the coarse grains,^[^ ^99^ ^]^ which is the reason that a longer elongation occurs at the low strain rates.

In the current results, cavities exert an important influence on the superplastic deformation of HC‐LRMEA at 1173 K and 10^−2^ s^−1^. The cavities within the coarse grains are relatively independent, which differ from those in the fine‐grain region (Figure 3i; Figure S13, Supporting Information). The growth of these cavities is restricted by the BCC matrix and the ultrafine particles, thereby avoiding premature fracture due to the rapid increase in cavity volume.^[^ ^19^ ^]^ The cavities are typically found near the grain boundaries in fine‐grained regions (Figure 3i; Figure S13, Supporting Information). The GBS is the dominant deformation mechanism in this region at the last stage of coarse‐grained superplasticity. A cavity damage caused by the GBS can be restricted by an interlinkage of cavity strings during plasticity‐controlled cavity growth.^[^ ^18^ ^]^ The Zr‐rich particles at the grain boundaries can block the cavity growth.^[^ ^100^, ^101^ ^]^ The microstructure of the present HC‐LRMEA manipulates the cavity interlinkage, which has a significant impact on maintaining quasi‐stable plastic flow.^[^ ^102^ ^]^

As discussed, this deformation process is denoted as a consecutively triggered “domino” superplastic mechanism (Figure S9, Supporting Information), including a plot of the distinct effects of the consecutive DS, DRX, and GBS mechanisms on the flow stress of the specimen with increasing specimen elongation. Note that the flow stress of the specimen decreases with increasing elongation in the stages corresponding to the three sequentially operative primary deformation mechanisms. Accordingly, this consecutive process facilitates the observed superplasticity in the coarse‐grained HC‐LRMEA material at a high strain rate.

## 3 Conclusions

In conclusion, the current study finds that a rare high‐strain rate and coarse‐grained superplasticity (>440%) achieved in a gigapascal LRMEA with a microstructure of ultrafine particles embedded in the BCC matrix. A consecutively triggered “domino” superplastic mechanism is found to elucidate the superplastic deformation behavior. Differing from the conventional coarse‐grained alloys deformation dominated by solute drag creep, this “domino” coarse‐grained superplastic deformation mechanism is a multideformation mechanism, including a sequence of dislocation sliding, dynamic recrystallization, and grain boundary sliding, which can be consecutively triggered, like dominoes. The detailed results of microstructural evolution analysis demonstrate DS inside the coarse grains at low specimen elongation, the production of fine‐grained particles via DRX at the coarse‐grain boundary areas under moderate specimen elongation, and GBS among the fine‐grained particles at superplastic elongation. During such coarse‐grained superplasticity, the ultrafine particles play significant roles to produce the high deformation stresses that are sufficient to overcome the solute drag effect, to promote dynamic recrystallization that can form the fine grain regions, and to enhance the strength of LRMEA, respectively.

The “domino” superplastic mechanism can be extended to other materials with similar structures, and can serve as a guide for designing novel high‐strength and coarse‐grained superplastic alloys (Figure S15, Supporting Information). Accordingly, through tuning the microstructure or chemical compositions of alloys, a dual‐phase microstructure, i.e., high‐density ultrafine particles uniformly dispersed in a solid solution matrix, must be formed, in which the matrix phase has a stable flow capacity, a relatively high activation energy to ensure the dislocation sliding controlled creep, and a high solid solution strengthening effect to help coarse‐grain yet high strength. Furthermore, the particles are required to be thermally stable. Thus, the “domino” coarse‐grained superplastic performance can be achieved without introducing grain refinement processes, making superplastic formation more economically viable for the manufacturing of complex, high‐quality components used in aerospace and biomedical engineering applications.

## 4 Experimental Section

### Sample Preparation and Mechanical Property Tests

The Ti_43.3_V_28_Zr_14_Nb_14_Mo_0.7_ (HC‐LRMEA) materials were arc melted to be alloy ingots under argon with high‐purity (99.95%) Ti, V, Zr, Nb, and Mo metal precursors. Each ingot was melted at least five times to acquire a uniform composition. Then, all ingots were formed into plate samples with dimensions of 10 × 80 × 5 mm by drop‐casting into a water‐cooled copper mold. The as‐cast plates were homogenized in a high‐temperature vacuum oven at 1273 K under – 0.5 Pa of atmospheric pressure for 0.5 h and then annealed at a temperature of 1173 K under vacuum conditions for 2 h, finally cooled down to room temperature in air.

The dog‐bone‐shaped specimens for tensile testing were fabricated by electrical discharge machining (EDM), with a gauge length of ≈6 mm parallel to the rolling direction, a width of ≈2 mm, and a thickness of ≈1 mm. The obtained specimens were coated with a ≈50‐µm‐thick Al_2_O_3_ film to prevent oxidation during tensile testing.

Tensile tests were conducted using an Instron 8801 universal testing machine equipped with a CP122021 furnace. The specimens were heated in the open air from room temperature to the target testing temperatures of 973 (0.45*T*_m_, *T*_m_ is the melting point), 1073 (0.5*T*_m_), and 1173 K (0.55*T*_m_), at a heating rate of ≈10 K min^−1^. Then, they were allowed to remain at the designated temperature for 5 min before testing to achieve a stable target temperature. Subsequently, the specimens were preloaded at 50 N to eliminate any sample elongation due to thermal expansion, and a tensile load was applied at a strain rate of 10^−3^ or 10^−2^ s^−1^, with sample elongation recorded at 1 s intervals until fracture. The fractured specimens were promptly immersed in water to preserve their microstructure. In addition, the obtained elongation values were confirmed according to the measurements of the fractured samples, which were obtained using an optical microscope. Three tests were conducted for each condition to ensure good reproducibility of the results.

The microhardness was measured on a Struers ApS standard Vickers microhardness tester using a 200 g load for 15 s. The average of a random 9 × 9 lattice from each sample was considered. The spacing between each test point was about 200 µm.

### Microstructural Characterization

The phase structures of the samples were analyzed by X‐ray diffraction (XRD) using a Rigaku D∖max 2550 diffractometer with Cu K*α* radiation (*λ* = 1.54056 Å) at 40 kV over a 2*θ* range of 20° to 100° with a scanning speed of 1° min^−1^. The microstructures were characterized by scanning electron microscopy (SEM) using a Shimadzu EPMA‐8050G microscope. The samples employed for SEM analysis were first mechanically polished using 3000‐grit SiC abrasive paper and then electro‐polished for 30 s via a twin‐jet polishing technique using a solution composed of 6 vol% perchloric acid, 34 vol% n‐butanol, and 60 vol% methanol at a temperature of −40 °C to thin the samples and remove residual stress. The elemental distributions on the sample surfaces were evaluated based on electron probe micro‐analyzer (EPMA) mapping analysis, which was conducted at a voltage of 15 kV and current of 200 nA with a beam size of 0.2 µm. Electron backscatter diffraction (EBSD) characterization was conducted using a CamScan Apollo 300 scanning electron microscope equipped with an HKL‐Technology EBSD system at a scanning step size of 0.5 µm. The samples used for EBSD analysis were prepared using the same process described above for SEM analysis. The average diameters of the grains were measured based on their area fractions, which were observed in the EBSD mapping results using Image‐Pro Plus software (package 5). The average values were obtained based on at least three images, depending on the number of visible grains in each image. The density of the geometrically necessary dislocations (*ρ*_GND_) was calculated by the following expression^[^ ^103^ ^]^

(2) $$\rho_{\mathrm{GND}}=2\mathrm{KA}M_{\mathrm{ave}}/\mu b$$

where KAM_ave_ is the kernel average misorientation, namely, *ρ*_GND_ = 2*θ*/*µb*,^[^ ^104^ ^]^ *θ* is the misorientation angle, *µ* is the unit length (*µ* = 10^−5^ m),^[^ ^103^ ^]^ and *b* is the magnitude of the Burgers vector.^[^ ^40^ ^]^ EBSD technique is also used to distinguish the DRX grains, according to the orientation difference between two adjacent grains can be increased due to the recrystallization, which can approach the large angular grain boundaries (>15°).^[^ ^105^, ^106^ ^]^ By counting the grain boundaries in selected representative regions based on the EBSD technique,^[^ ^107^ ^]^ the number of recrystallization of the material can be qualitatively analyzed and the recrystallized grains can be distinguished.

Transmission electron microscopy (TEM) imaging was carried out using a JEM‐2100F microscope at 200 kV and an energy dispersive spectrometer (EDS) from Oxford Instruments. The samples employed for TEM analysis were cut from the samples both before and after fracture under tensile stress testing using a diamond saw. Then, the samples were ground down to a thickness of ≈30 µm. Finally, the mentioned twin‐jet polishing technique was applied to prepare the electron‐transparent TEM foils.

## Conflict of Interest

The authors declare no conflict of interest.

## Supporting information

Supporting Information

## Data Availability

Research data are not shared.
