# Supplementary material for: Efficient Coarse‐Grained Superplasticity of a Gigapascal Lightweight Refractory Medium Entropy Alloy
Source: Adv Sci (Weinh). 2023 Feb 19;10(12):2207535. doi: 10.1002/advs.202207535 (PMC10131849; doi:10.1002/advs.202207535)
Supplement: Supplementary file 1 — Supporting Information [file ADVS-10-2207535-s001.pdf]

ADVANCED SCIENCE
Open Access

# Supporting Information

for *Adv. Sci.*, DOI 10.1002/advs.202207535

Efficient Coarse-Grained Superplasticity of a Gigapascal Lightweight Refractory Medium Entropy Alloy

*Yuefei Jia, Shiwei Wu, Yongkun Mu, Long Xu, Chang Ren, Kang Sun*, Jun Yi, Yandong Jia, Wentao Yan and Gang Wang**

Supporting Information

# Efficient Coarse-grained Superplasticity of a Gigapascal Lightweight Refractory Medium Entropy Alloy

*Yuefei Jia*[1,2,3], *Shiwei Wu*[3], *Yongkun Mu*[1,2], *Long Xu*[1,2,3], *Chang Ren*[1,2], *Kang Sun*[1,2,*], *Jun Yi*[1,2], *Yandong Jia*[1,2], *Wentao Yan*[3], *Gang Wang*[1,2,*]

1 Institute of Materials, Shanghai University, Shanghai 200444, China

2 Zhejiang Institute of Advanced Materials, Shanghai University, Jiashan 314100, China

3 Department of Mechanical Engineering, National University of Singapore, 117575, Singapore

*Email: sunkang@shu.edu.cn and g.wang@shu.edu.cn

Content:

1. Supplementary Text
2. FigureS1: Activation energy of the HC-LRMEA at deformation strains of ~20%, ~100%, and ~300%.
3. FigureS2: Microstructural characterization after superplastic deformation at 1173 K.
4. Figure S3: Elemental distribution during the superplastic deformed sample at 1173 K.
5. Figure S4: Tensile performance.
6. Figure S5: The mean geometrically necessary dislocations (GND) for different areas.
7. Figure S6: The volume fraction and mean grain size of the DRX grains.
8. Figure S7: TEM Characteristics of the superplastic deformed specimen of CH-LRMEA at 1173 K.
9. Figure S8: The dislocations and subgrains observed in the superplastic deformed specimen at 1173 K.
10. Figure S9: Schematic illustrating the consecutively triggered deformation mechanism operative in the coarse-grained HC-LRMEA specimens.
11. Figure S11: EBSD characterizations and schematic illustrations of the superplastic deformation.

12. Figure S12: Microstructure analysis after superplastic deformation of HC-LRMEA.
13. Figure S13: Flow stress-superplastic strain curves and strain rate sensitivities of HC-LRMEA
14. Figure S14: Fracture morphology after the superplastic deformation of HC-LRMEA at 1173 K.
15. Figure S15: Inverse design schematic of lightweight, high-strength, and coarse-grained superplastic alloys.
16. Table S1: The mechanical properties of the coarse-grained superplastic alloys.

**Supplementary Text**

**1.1. Activation Energy**

High-temperature deformation or creep is a thermally activated process. In general, the Zener Hollomon parameter Z(σ) can describe the relationship between temperature and flow stress at each strain rate [1]. The formula of Z(σ) is given by:

$$Z(\sigma) = A\sigma^{n} = \dot{\varepsilon}\, exp(\,Q/RT), \quad (2)$$

where Q is the activation energy, n is the stress exponent, A is a constant sensitivity to the deformation mechanism, and R is the gas constant. Q can be determined according to the following relations:

$$Q = -R\frac{\partial\, ln\, \dot{\varepsilon}}{\partial(1/T)}\bigg|_{\sigma} = nR\frac{\partial\, ln\, \sigma}{\partial(1/T)}\bigg|_{\dot{\varepsilon}}. \quad (3)$$

Figure S1a shows the plotted log$\sigma$ versus 1000/T curves, and the value of Q is also calculated from the slope of the curves. The activation energy (Q) is about 234.2 to 314.6 kJ/mol with the tensile strain from 20% to 300% at 1173 K with a strain rate of $10^{-2}$ $s^{-1}$. According to the sluggish effect [2] in HEA, the diffusion coefficient is likely to be controlled by the slowest moving species, namely β-Ti in our HC-LRMEA. Figure S1b shows that the value of the activation energy ($Q_{20\%}$) is below $Q_{\beta\text{-Ti}}$ at Stage I. At Stage II, $Q_{100\%}$ is similar to $Q_{\beta\ Ti}$. Finally, the value of $_{300\%}$ is larger than $Q_{\beta\text{-Ti}}$ at Stage III. The potential mechanisms of superplastic deformation will be discussed in the discussion section.

**1.2. Microstructure after Superplasticity**

Figure S2 presents the microstructural characteristics of the HC-LRMEA after superplastic deformation at 1173 K. A notable phenomenon pertains to the occurrence of dynamic recrystallization (DRX) at the coarse grain boundaries. Specifically, Figure S2a shows the EBSD IPF image exhibiting significant elongation of the coarse grains by about

100–200 μm due to the superplastic deformation and presence of numerous fine grains at the coarse grain boundaries, resulting from DRX. The fine grains of DRX, 1~10 μm in size, are evident between the coarse grains, as illustrated in Figure S1b, and the volume fraction of the fine grains is about 16.5%. Figure S2c presents the TEM-BF image of the grain interior displaying both bright and dark phases. As shown in Figure S2d, the corresponding selected area electron diffraction along the [001] direction confirms that the bright area is the BCC matrix, while the dark area is comprised of the Zr-rich particles. Figure S2e displays a large number of Zr-rich particles embedded within the grain and grain boundary. The corresponding EDS mappings of Ti, V, Zr, Nb, and Mo revealed the distribution of elements in this region, with Ti, V, Nb, and Mo enriched in the intragrain matrix, but at lower content at the grain boundary. By contrast, Zr is enriched in the precipitated phase and at the grain boundary. In addition, the size of the Zr-rich particles increases to 500 ~1000 nm relative to the initial state, and the volume fraction of this phase also increases to approximately 40%.

**1.3. Grain Boundary Segregation**

The phenomenon of grain boundary segregation is observed in the current superplastic deformation at 1173 K, which is regarded as an active factor in inducing DRX at the coarse-grained boundaries. Figure S3a presents the elongated coarse grains along the tension direction. An area selected for EPMA analysis near the grain boundaries exhibits an almost uniform distribution of Ti, Nb, and Mo (Figure S4c). However, there is a significant separation between Zr and V. In particular, large atomic size Zr elements tend to concentrate along the tension direction toward the grain boundaries. By contrast, the small atomic size V elements tend to diffuse inward of the coarse grains. Moreover, the Zr elements eventually form a Zr-rich segregation layer with a thickness of about 200 to 300 nm, or with large amounts of Zr-rich particles at the grain boundaries (Figure S3b).

Based on thermodynamic calculations, the mixing enthalpies of V with the other two host elements (Ti, Nb) are significantly skewed to negative values, namely $\Delta H_{Ti\text{-}V}=-2$ kJ/mol and $\Delta H_{Nb\text{-}V}=-1$ kJ/mol, which suggest that V tend to combine with Ti and Nb. By contrast, the enthalpies of mixing Zr with Ti and Nb are $\Delta H_{Ti\text{-}Zr}=0$ kJ/mol and $\Delta H_{Nb\text{-}Zr}=4$ kJ/mol, respectively, which implies that Zr tends to exist independently in this Ti-V-Zr-Nb multiple systems. In addition, the atomic radii of V and Zr are 135 and 155 pm [3], respectively, with a size mismatch of 6.9%, which was one of the main factors contributing to the separation of the two elements. In addition, lattice distortion contributed to enhancing the diffusion of Zr in the RHEA[4]. Thus, under rheological stress and temperature, the Zr elements tend to move into the high-energy zone at the grain boundary. The diffusion and segregation of Zr elements

play a dual role, as they promote DRX at the coarse grain boundaries; however, they also drove GBS, which, in turn, promotes superplastic deformation, which has also been found in other high-strain-rate superplastic medium entropy alloys [5].

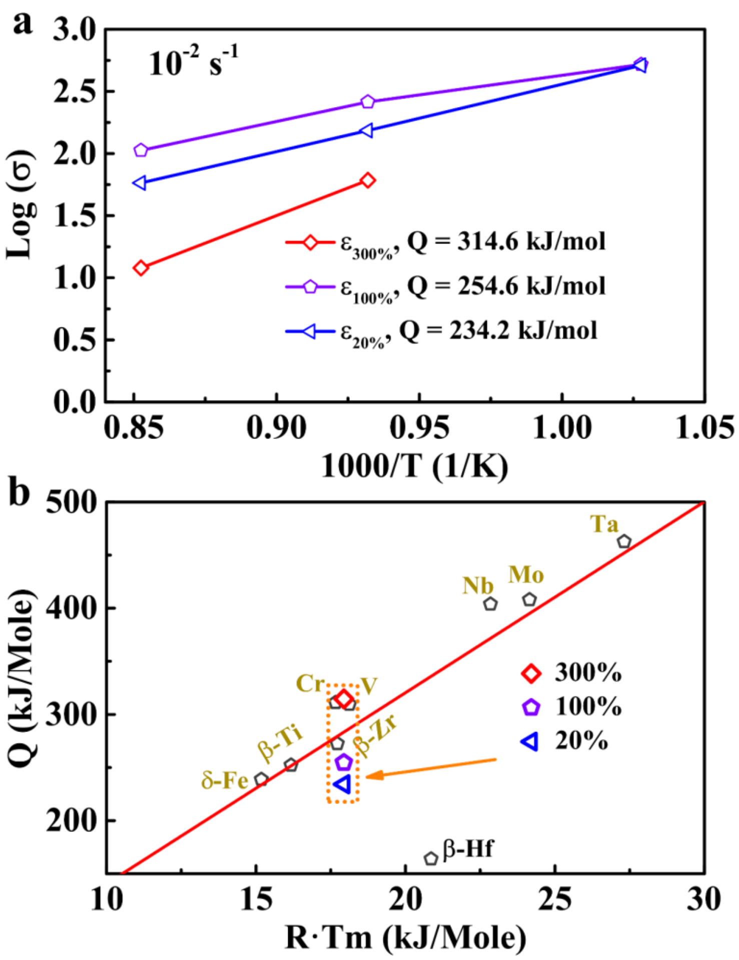


**Figure S1.** Activation energy of the HC-LRMEA at deformation strains of ~20%, ~100%, and ~300%. (a) log-scale true stresses (σ) as a function of the inverse absolute temperature at true strains of 20%, 100%, and 300%, where the slopes of these linear dependences determine the activation energies (Q); (b) comparison of activation energies of pure BCC metals and HC-LRMEA.

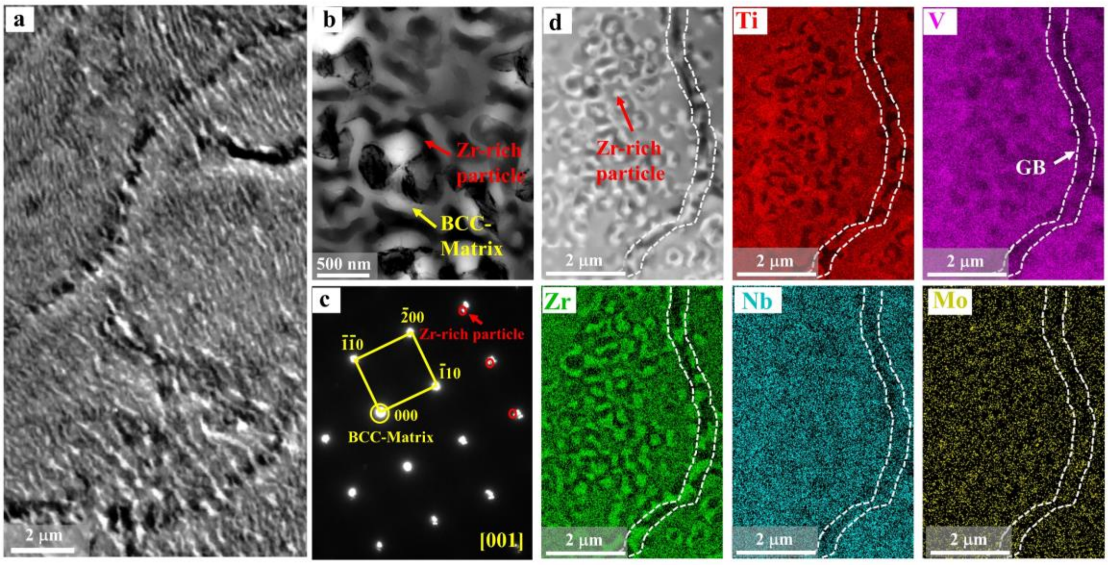


**Figure S2.** Microstructural characterization after superplastic deformation at 1173 K. (a) SEM image; (b) TEM-bright field image exhibiting the morphology and distribution of Zr-rich particle; (c) the corresponding SAED pattern; (d) STEM image and corresponding EDS mapping of Ti, V, Zr, Nb, and Mo.

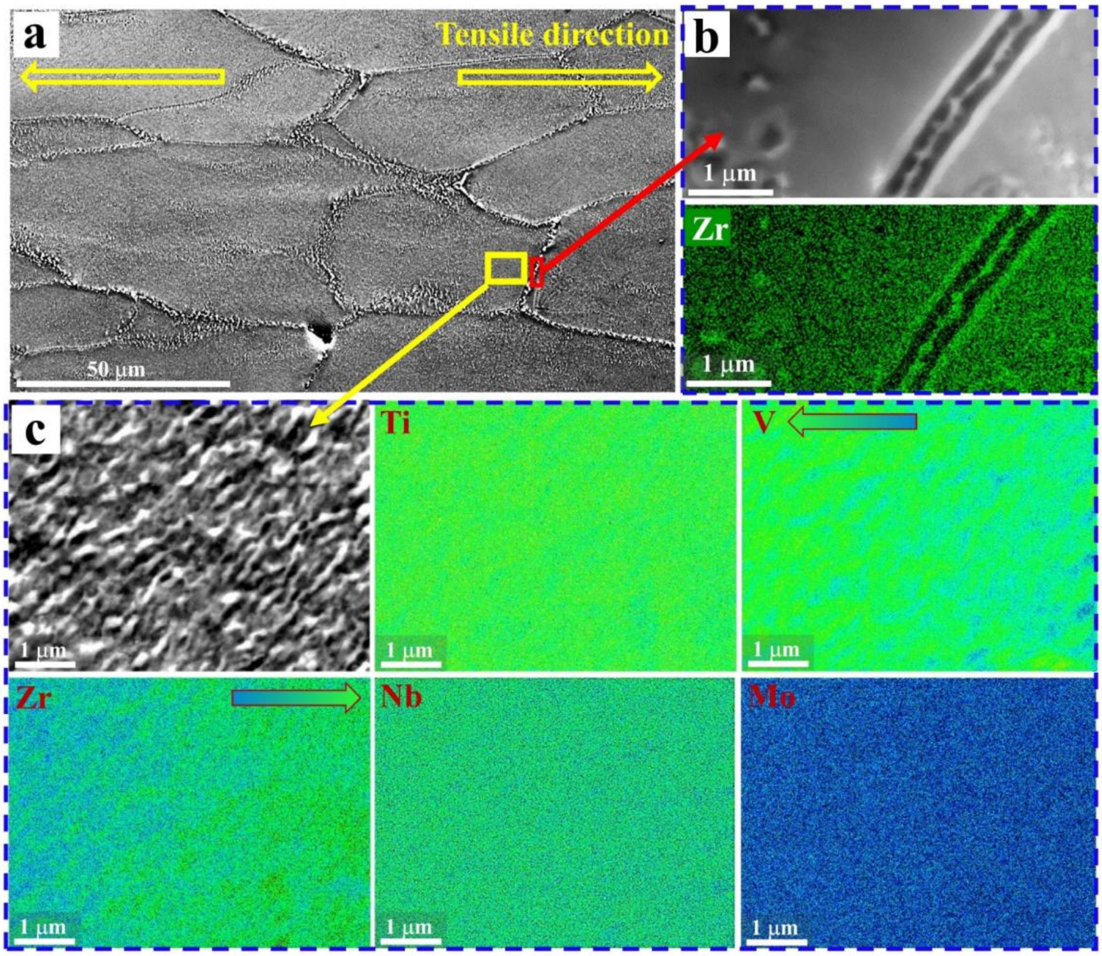


**Figure S3.** Elemental distribution during the superplastic deformed sample at 1173 K. (a) SEM image of the sample during the deformed state along the tensile direction; (b) STEM and corresponding EDS map showing a concentration of Zr at the grain boundary; (c) SEM and corresponding EPMA mapping showing the distribution of Ti, V, Zr, Nb, and Mo.

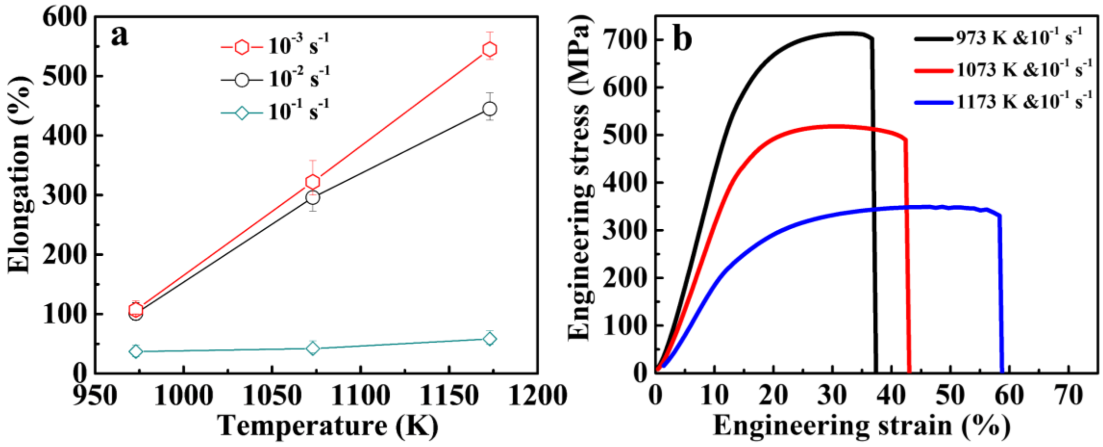


**Figure S4.** Tensile performance. (a) the total elongation vs temperature under different strain rates of $10^{-2}$ $s^{-1}$ and $10^{-3}$ $s^{-1}$; (b) engineering stress and strain curves at 973 K, 1073 K and 1173 K at strain rate of $10^{-1}$ $s^{-1}$.

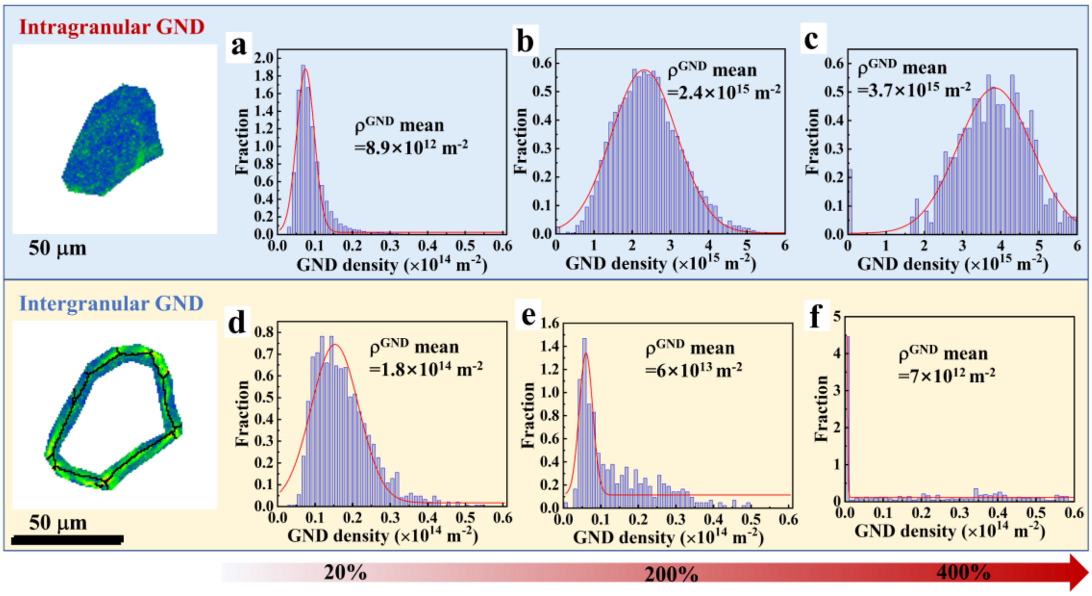


**Figure S5.** The mean geometrically necessary dislocations (GND) for different areas. (a), (b), and (c) indicating the intragranular mean GND density distributions; (d), (e) and (f) exhibiting the intergranular mean GND density distributions, (a) and (d) at the strain of ~20%; (b) and (e) at the strain of ~200%; (c) and (f) at the strain of ~ 400%.

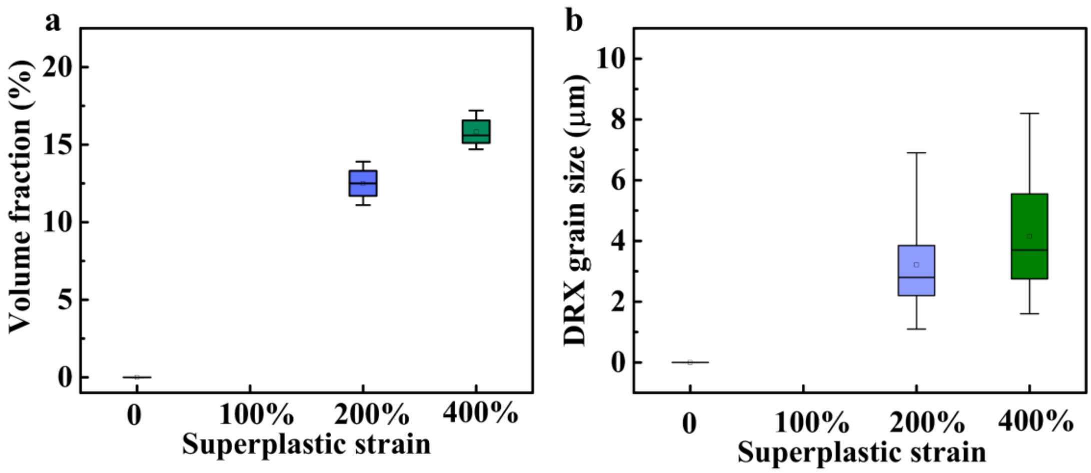


**Figure S6.** The volume fraction (a) and mean grain size (b) of the DRX grains produced in the superplastic process at superplastic strains of ~20%, ~200%, and ~400%.

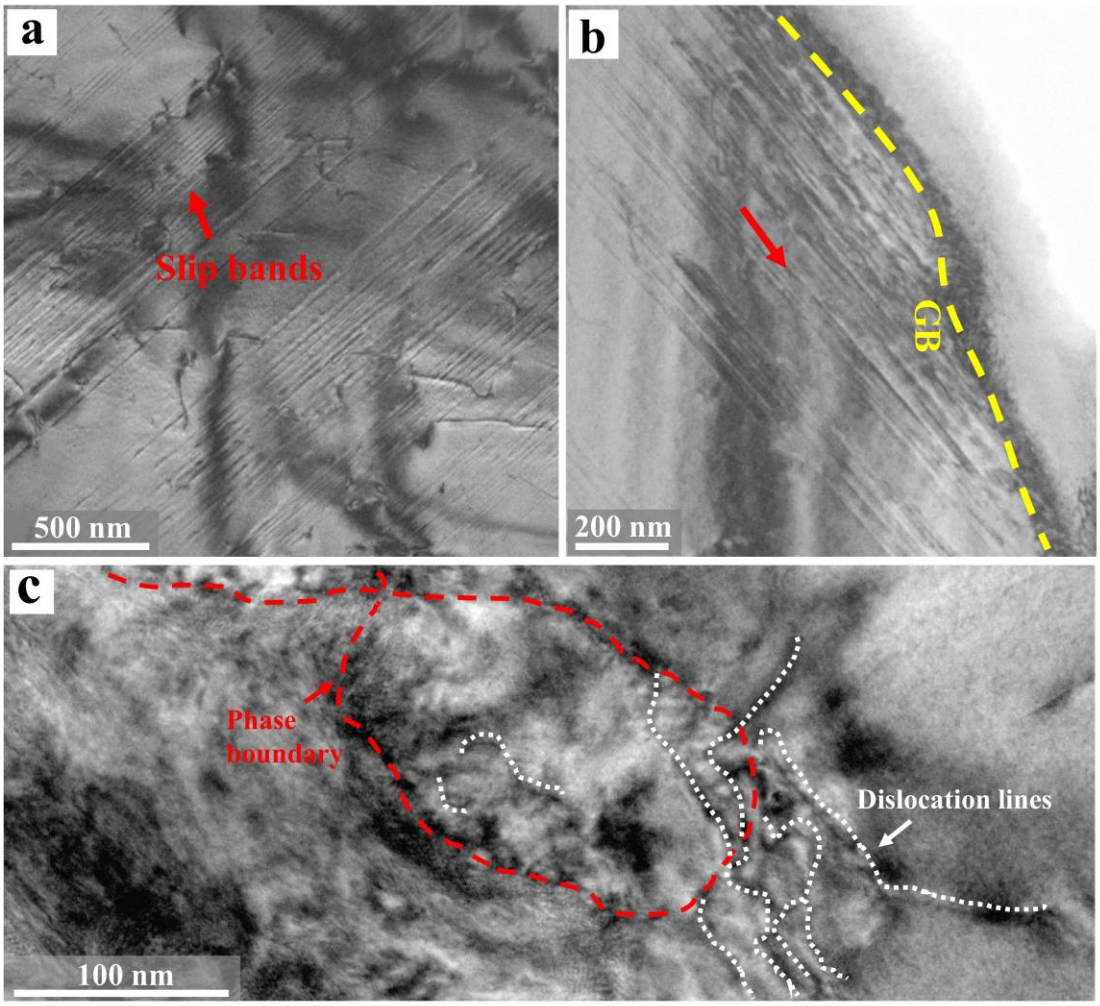


**Figure S7.** TEM Characteristics of the superplastic deformed specimen of CH-LRMEA at 1173 K. (a) TEM-BF image showing the slip bands inner the grain; (b) TEM-BF image indicating the slip bands near the grain boundary; (c) TEM-BF image presenting the deformed Zr-rich particle.

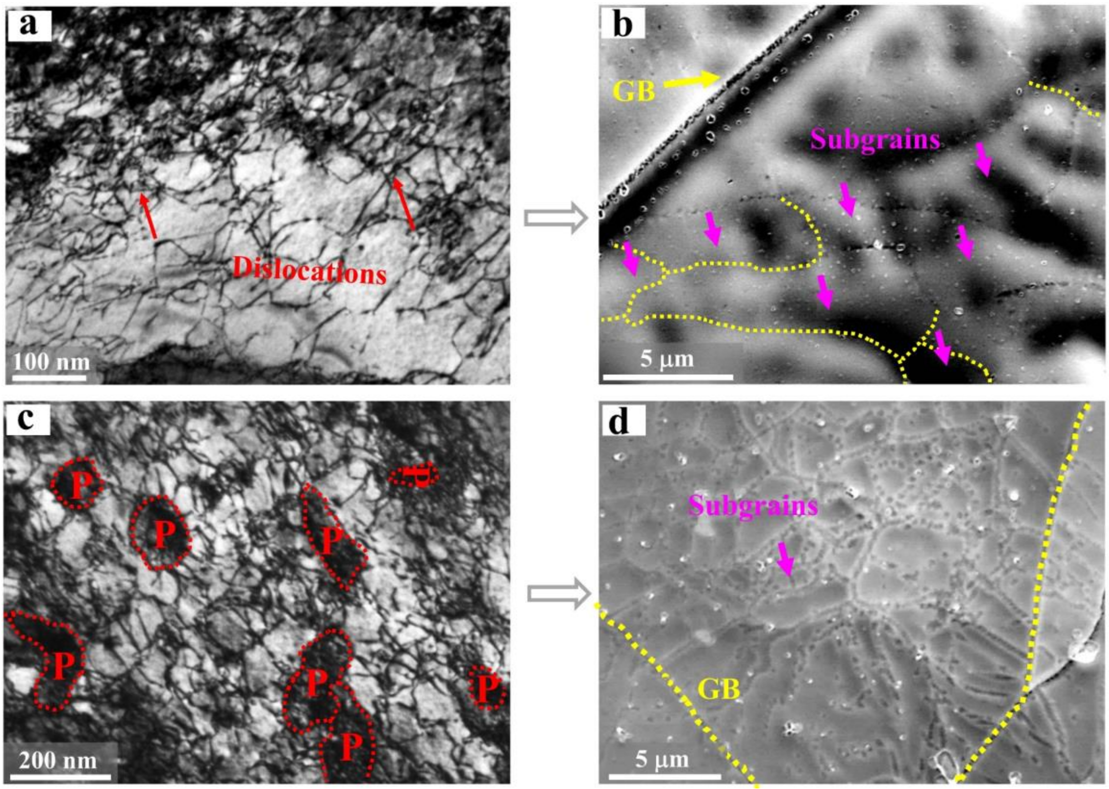


**Figure S8.** The dislocations and subgrains observed in the superplastic deformed specimen at 1173 K. (a) TEM-bright field image showing the deformation-induced dislocations; (b) SEM image exhibiting the subgrains inner the deformed coarse grain; (c) dislocations at the second-phase particles observed by TEM; (d) SEM image indicating the formation of subgrains.

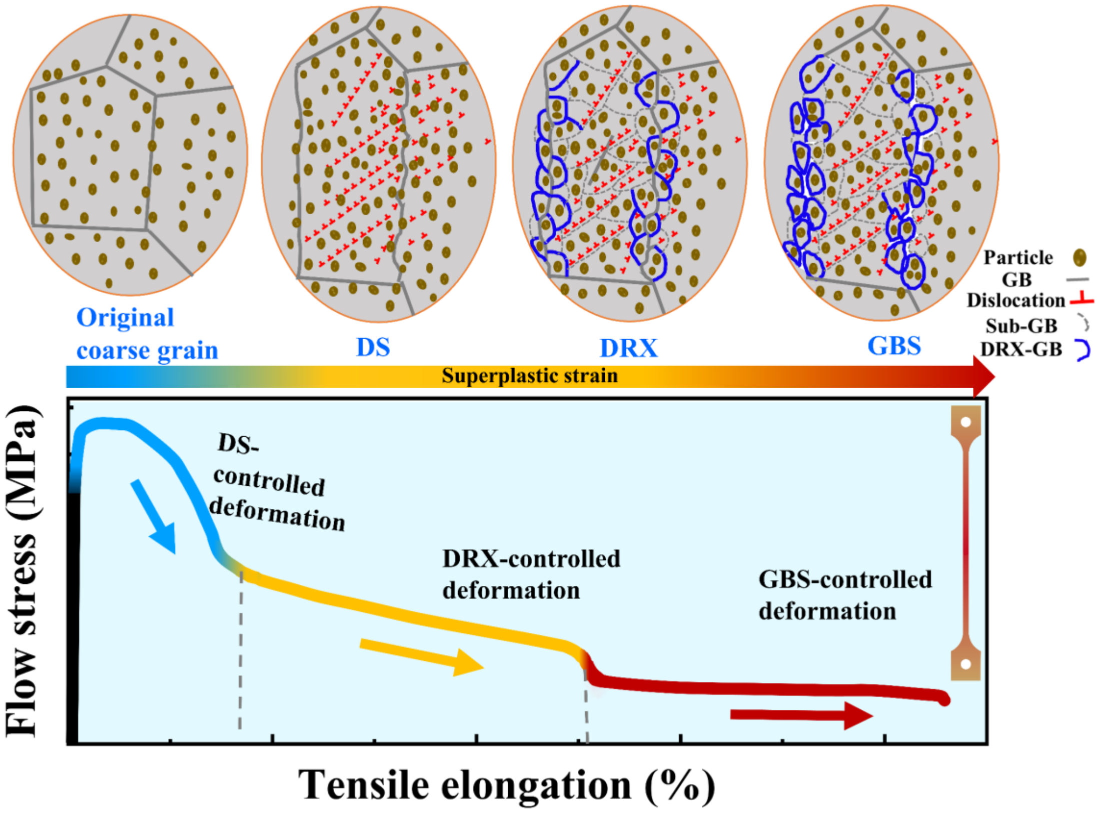


**Figure S9.** Schematic illustrating the consecutively triggered deformation mechanism operative in the coarse-grained HC-LRMEA specimens. The CTDM was composed of three primary deformation mechanisms that were individually operative within different ranges of tensile elongation and had distinct effects on the flow stress of the specimen. These consecutive mechanisms included dislocation sliding (DS), DRX, and GBS. "GB" representing grain boundary; "sub-GB" representing subgrain boundary; "DRX-GB" representing DRX grain boundary.

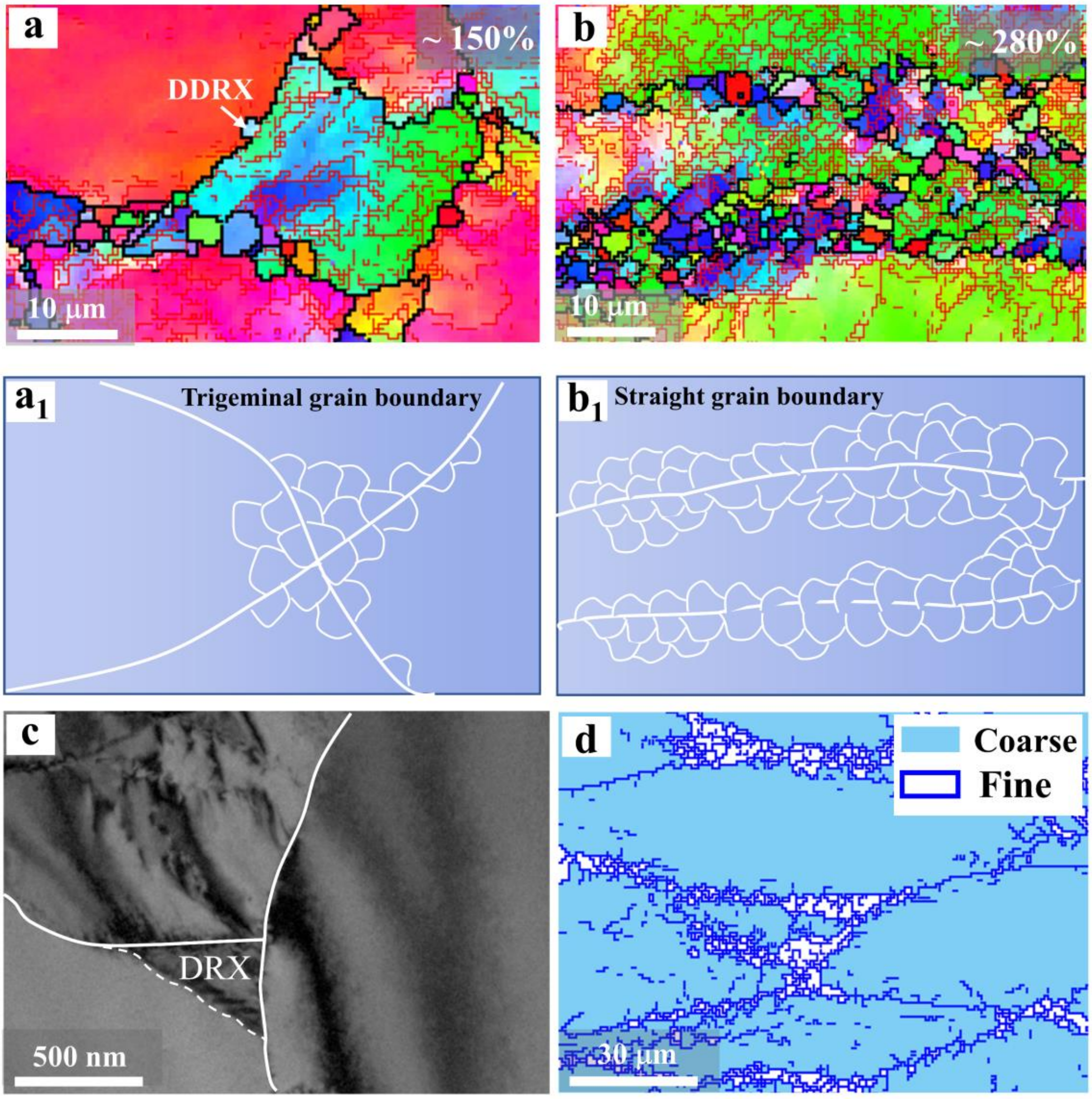


**Figure S10.** Microstructures and schematic illustrations of the superplastic deformation. (a) and (b) are EBSD IPF maps presenting DRX fine grains at a tensile strain of ~150% and ~280%; (a1) and (b1) are the corresponding schematic illustrations; (c) is TEM BF image showing a trigeminal grain boundary; (d) EBSD recrystallization image showing the mixing of fine DRX grains and coarse grains.

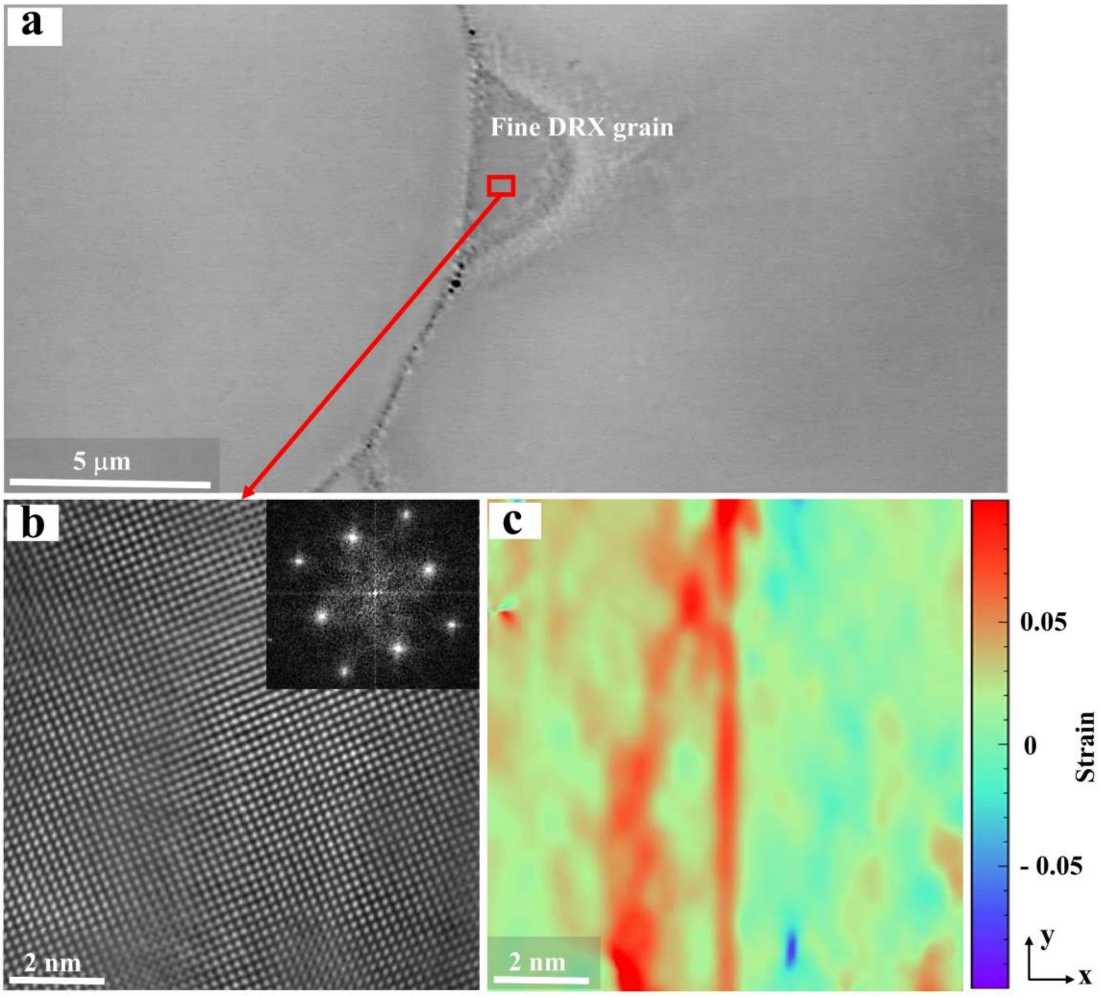


**Figure S11.** Microstructure analysis after superplastic deformation of HC-LRMEA. (a) SEM image showing a fine DRX grain at the coarse-grained boundary; (b) HRTEM of the DRX grain showing the deformed lattice structure; (c) the corresponding strain map of (b).

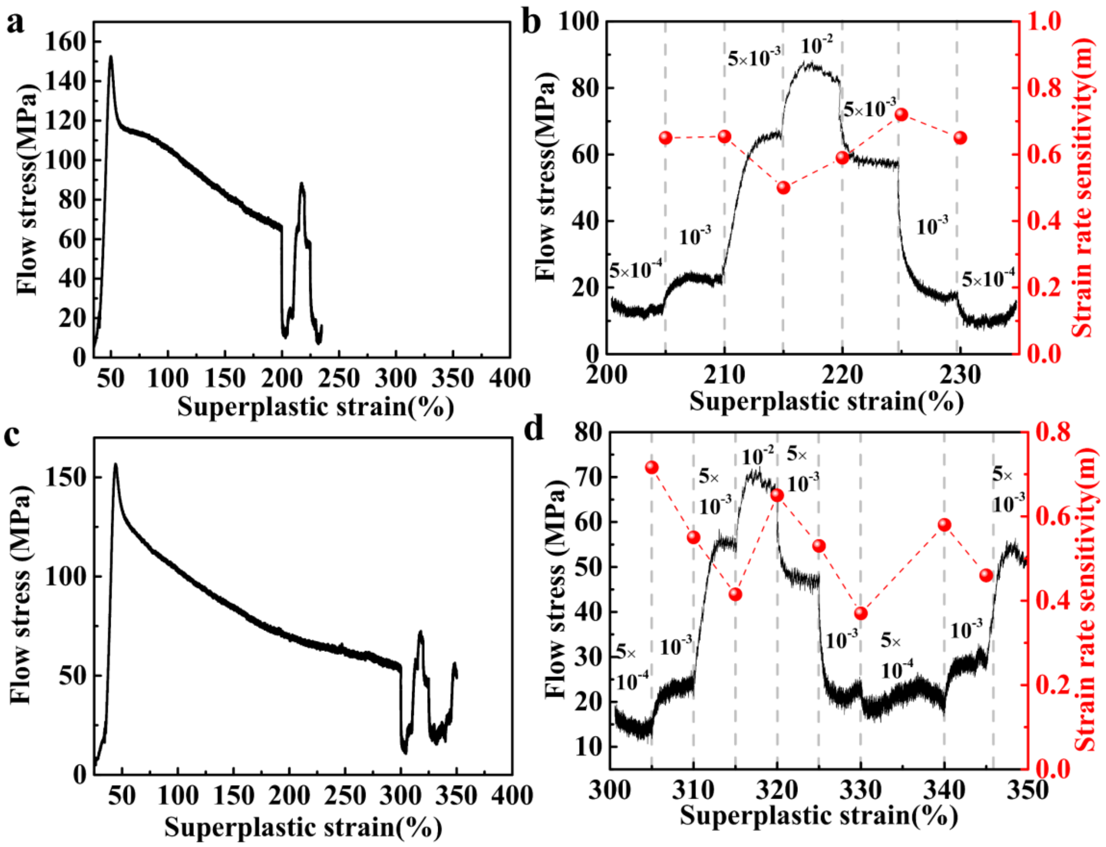


**Figure S12.** Flow stress-superplastic strain curves and strain rate sensitivities of HC-LRMEA. (a) flow stress- superplastic strain curve with a strain rate of $10^{-2}s^{-1}$ at 1173 K when the tensile strain <200% and with a jump test at 200 ~250%. (b) the jump strain rate curves and the corresponding strain rate sensitivity at the superplastic strain of 200 ~250%. (c) flow stress-superplastic strain curve with a strain rate of $10^{-2}$ $s^{-1}$ at 1173 K when the tensile strain <300% and with a jump test at 300 ~350%. (d) the jump strain rate curves and the corresponding strain rate sensitivity at the superplastic strain of 300 ~350%.

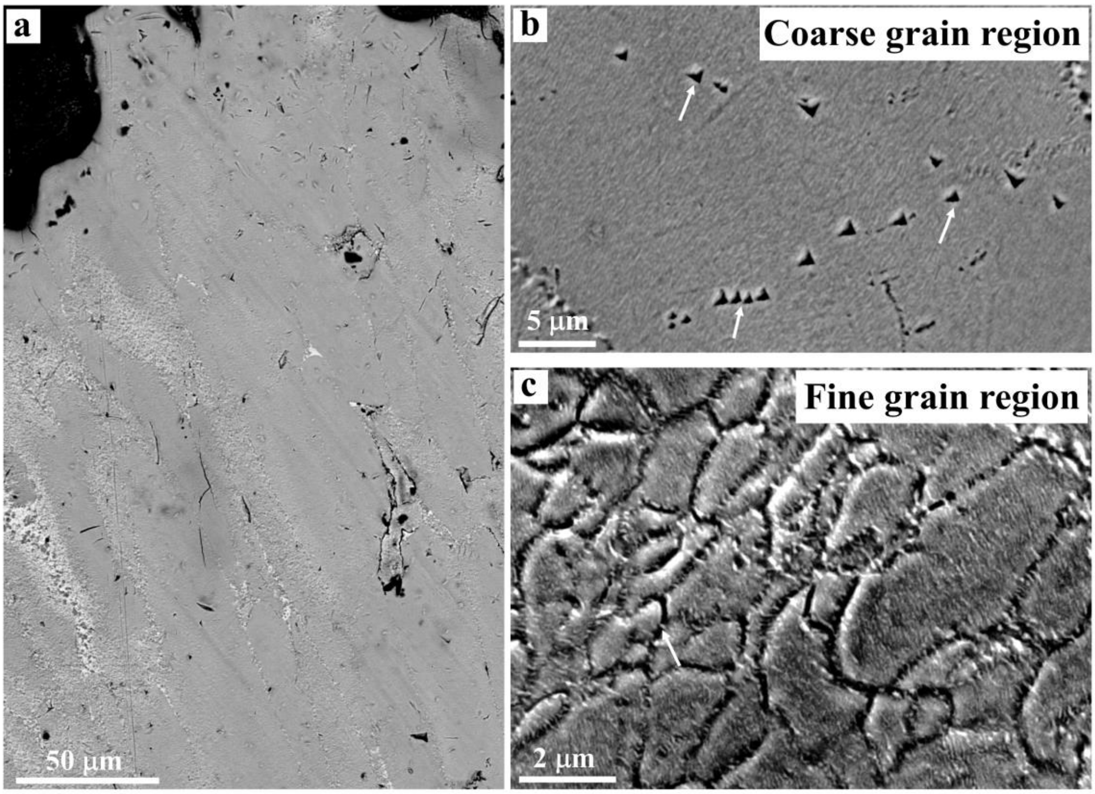


**Figure S13.** SEM characterization of superplastic deformation induced cavities of HC-LRMEA. (a) SEM image near the fractured part; (b) the distribution of the cavities in the coarse grain; (c) the distribution of the cavities in the fine grain region.

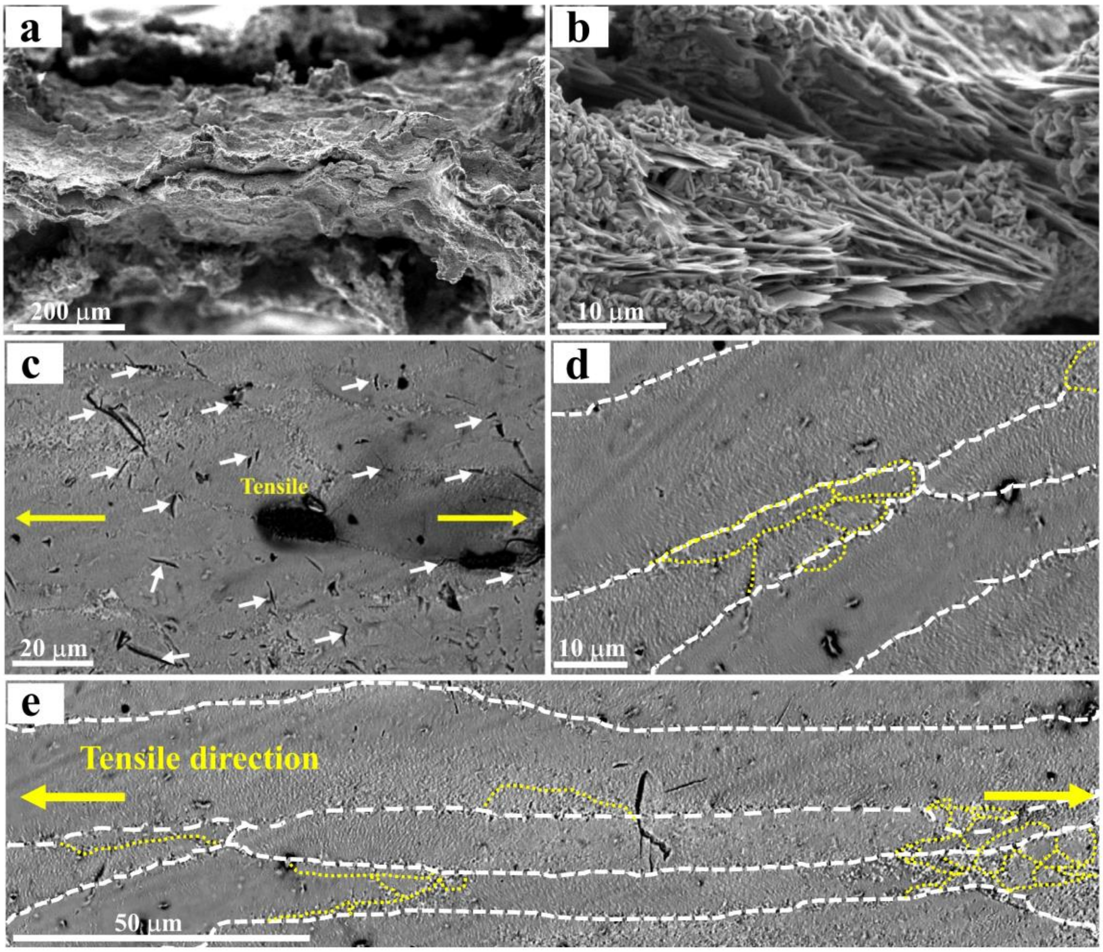


**Figure S14.** Fracture morphology after the superplastic deformation of HC-LRMEA at 1173 K. **(a)** SEM image showing the fractured morphology after superplastic deformation; (**b**) High magnification SEM image showing the fractured characterization; (**c**) Microcrack distribution near the fractured end; (**d**) SEM image near the fracture end showing the dynamic recrystallization; (**e**) Elongated grains and DRX grains during superplastic deformation.

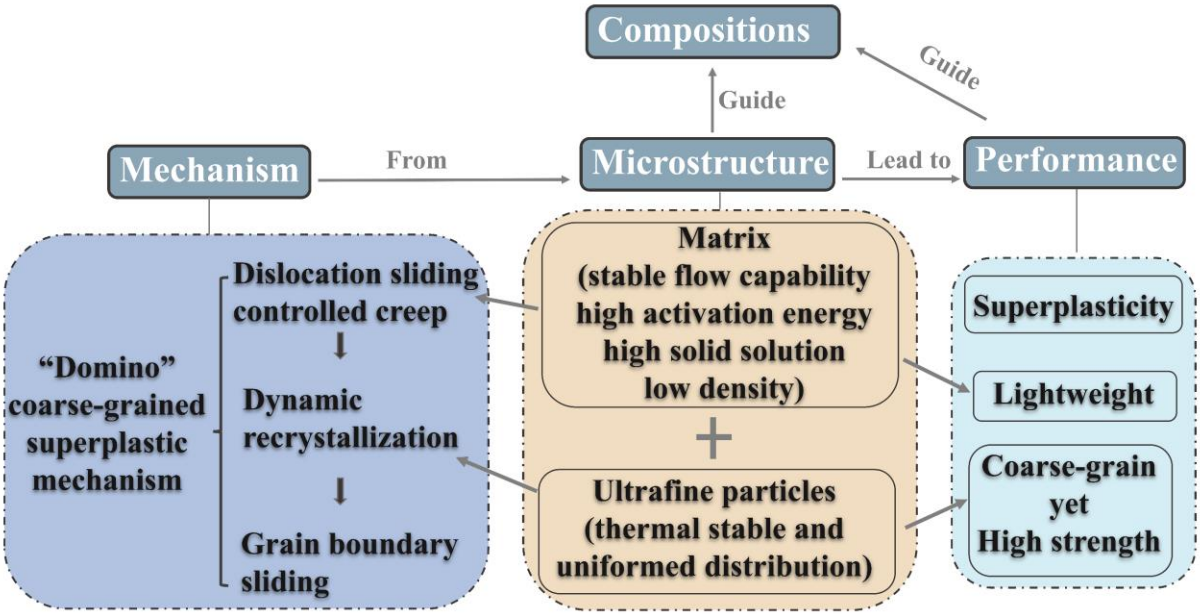


**Figure S15.** Inverse design schematic of lightweight, high-strength, and coarse-grained superplastic alloys.

Table S1. The mechanical properties of the coarse-grained superplastic alloys

| Alloy | | YS (MPa) | ELRT (%) | ELHT ($10^{-2}$ $s^{-1}$) | ELHT ($10^{-3}$ $s^{-1}$) |
|---|---|---|---|---|---|
| | Mg-Li Alloy [6] | 160 | 26 | 187 | 344 |
| Mg alloys | Mg-9Al-1Zn [7] | 133 | 5 | - | 604 |
| | AZ31 [8] | 131 | 18 | 255 | 320 |
| | Al-11Mg [9] | 150 | - | 225 | 225 |
| | Al-7Mg [9-10] | 399 | - | 340 | 250 |
| | Al-5.3Mg [9] | 280 | - | 260 | 240 |
| Al alloys | Al-3.0Mg-0.25Mn [11] | 100 | - | 140 | 182 |
| | Al-Mg-Cu [12] | 190 | - | 260 | 320 |
| | Al-4.5Mg-0.09Si [13] | 130 | - | - | 360 |
| | Al-Mg-Zn [14] | 160 | - | 400 | 375 |
| | 7475 [15] | 462 | - | 280 | 360 |
| | CP-Ti [16] | 290 | 20 | 160 | 190 |
| α-Ti alloys | Ti-6Al-2Sn-4Zr-2Mo-0.1Si [17] | 830 | 8 | 382 | 468 |
| | Ti-6Al-4V [18] | 1030 | 8 | 220 | 300 |
| | Ti-6Al-6V-2Sn [19] | 1020 | - | 242 | - |
| | Beta C [20] | 1280 | 10 | - | 101 |
| β-Ti alloys | Ti-13V-11Cr-3Al [21] | 1207 | - | 200 | 200 |
| | Ti-15V-3Cr-3Sn-3Al [22] | 1035 | 6 | 260 | 300 |
| | Ti-10V-2Fe-3Al [23] | 1220 | 5.5 | 200 | 380 |
| | Ti-25V-15Cr-0.2Si [24] | 1025 | 10 | 290 | 300 |
| Niobium alloy | C103 [25] | 308 | 33 | - | 125 |
| Intermetallic alloys | Ti-47Al-2Mn-2Nb-1B [26] | 540 | 0.8 | - | 235 |
| | Fe3Al-Ti [27] | 520 | | | 180 |
| LRMEA (this work) | HC-LRMEA | 1045 | 22 | 440 | 548 |

## Supplemental references


[1] A. K. Mukherjee, J. E. Bird, J. E. Dorn, *Mater. Sci.* **1968** .

[2] K. Y. Tsai, M. H. Tsai, J. W. Yeh, *Acta Mater.* **2013**, *61* (13), 4887.

[3] D. B. Miracle, O. N. Senkov, *Acta Mater.* **2017**, *122*, 448.

[4] J. Zhang, C. Gadelmeier, S. Sen, R. Wang, X. Zhang, Y. Zhong, U. Glatzel, B. Grabowski, G. Wilde, S. V. Divinski, *Acta Mater.* **2022**, *233*.

[5] P. Asghari-Rad, N. T.-C. Nguyen, A. Zargaran, P. Sathiyamoorthi, H. S. Kim, *Scr. Mater.* **2022**, *207*.

[6] K. Lin, Z. Kang, Q. Fang, J. Zhang, *Adv Eng Mater* **2014**, *16* (4), 381.

[7] T. Mohri, M. Mabuchi, M. Nakamura, T. Asahina, H. Iwasaki, T. Aizawa, K. Higashi, *Mater. Sci. Eng. A* **2000**, *290* (1-2), 139.

[8] A. Bussiba, A. Ben Artzy, A. Shtechman, S. Ifergan, M. Kupiec, *Mater. Sci. Eng. A* **2001**, *302* (1), 56.

[9] S. S. Woo, Y. R. Kim, D. H. Shin, W. J. Kim, *Scr. Mater.* **1997**, *37* (9), 1351.

[10] M. Zha, Y. Li, R. H. Mathiesen, R. Bjørge, H. J. Roven, *Acta Mater.* **2015**, *84*, 42.

[11] E. M. Taleff, G. A. Henshall, T. G. Nieh, D. R. Lesuer, J. Wadsworth, *Metall Mater Trans A* **1998**, *29* (13), 1081.

[12] A. R. Chezan, J. T. M. De Hosson, *Mater. Sci. Eng. A* **2005**, *410-411*, 120.

[13] H. Hosokawa, H. Iwasaki, T. Mori, M. Mabuchi, T. Tagata, K. Higashi, *Acta Mater.* **1999**, *47* (6), 1859.

[14] M. A. García-Bernal, D. Hernandez-Silva, V. Sauce-Rangel, *J. Mater. Sci.* **2007**, *42* (11), 3958.

[15] D. H. Shin, Y. J. Joo, W. J. Kim, C. S. Lee, *J. Mater. Sci.* **1998**, *33* (12), 3073.

[16] X. J. Zhu, M. J. Tan, K. M. Liew, *Mater. Sci. Forum.* **2007**, *551-552*, 373.

[17] C. H. Park, B. Lee, S. L. Semiatin, C. S. Lee, *Mater. Sci. Eng. A* **2010**, *527* (20), 5203.

[18] E. Alabort, D. Putman, R. C. Reed, *Acta Mater.* **2015**, *95*, 428.

[19] M. Li, X. Liu, A. J. J. o. M. P. T. Xiong, **2002**, *121* (1), 1.

[20] G. C. Morgan, C. Hammond, *Mater. Sci. Eng.* **1987**, *86*, 159.

[21] A. H. Sheikhali, M. Morakkabati, S. M. Abbasi, A. Rezaei, *Int J Eng Sci* **2013**, *104* (11), 1122.

[22] M. Tan, X. Zhu, S. Thiruvarudchelvan, K. J. A. o. M. S. Liew, Engineering, **2007**, *28* (12), 717.

[23] Z. L. Zhao, H. Z. Guo, C. Y. Duan, Z. K. Yao, *Mater. Sci. Technol.* **2013**, *25* (4), 511.

[24] X. Zhang, Y. Zhao, W. Zeng, *Mater. Sci. Eng. A* **2010**, *527* (15), 3489.

[25] J. Wadsworth, C. A. Roberts, E. H. Rennhack, *J. Mater. Sci.* **1982**, *17* (9), 2539.

[26] D. Lin, F. Sun, *Intermetallics* **2004**, *12* (7-9), 875.

[27] A. Shan, D. Lin, M. Chen, *Chinese Science Abstracts Series A* **1995**, 64.
